# Toward the Understanding of Topographical and Spectral Signatures of Infant Movement Artifacts in Naturalistic EEG

**DOI:** 10.3389/fnins.2020.00352

**Published:** 2020-04-28

**Authors:** Stanimira Georgieva, Suzannah Lester, Valdas Noreika, Meryem Nazli Yilmaz, Sam Wass, Victoria Leong

**Affiliations:** ^1^Department of Psychology, University of Cambridge, Cambridge, United Kingdom; ^2^Department of Psychology, University of East London, London, United Kingdom; ^3^Division of Psychology, Nanyang Technological University, Singapore, Singapore

**Keywords:** electroencephalography, signal distortion, motion artifacts, infants, naturalistic paradigm

## Abstract

Electroencephalography (EEG) is perhaps the most widely used brain-imaging technique for pediatric populations. However, EEG signals are prone to distortion by motion. Compared to adults, infants’ motion is both more frequent and less stereotypical yet motion effects on the infant EEG signal are largely undocumented. Here, we present a systematic assessment of naturalistic motion effects on the infant EEG signal. EEG recordings were performed with 14 infants (12 analyzed) who passively watched movies whilst spontaneously producing periods of bodily movement and rest. Each infant produced an average of 38.3 s (SD = 14.7 s) of rest and 18.8 s (SD = 17.9 s) of single motion segments for the final analysis. Five types of infant motions were analyzed: Jaw movements, and Limb movements of the Hand, Arm, Foot, and Leg. Significant movement-related distortions of the EEG signal were detected using cluster-based permutation analysis. This analysis revealed that, relative to resting state, infants’ Jaw and Arm movements produced significant *increases* in beta (∼15 Hz) power, particularly over peripheral sites. Jaw movements produced more anteriorly located effects than Arm movements, which were most pronounced over posterior parietal and occipital sites. The cluster analysis also revealed trends toward *decreased* power in the theta and alpha bands observed over central topographies for all motion types. However, given the very limited quantity of infant data in this study, caution is recommended in interpreting these findings before subsequent replications are conducted. Nonetheless, this work is an important first step to inform future development of methods for addressing EEG motion-related artifacts. This work also supports wider use of naturalistic paradigms in social and developmental neuroscience.

## Introduction

### Motion in EEG Measurements

Electroencephalography (EEG) is a widely used brain imaging technique for both adult and pediatric populations, owing to its low risk to the individual (Teplan, 2002) and ease of application (De Haan, 2013). In particular, rising interest in the neural processes that play a critical part in early emotional, social, and cognitive development has led to an increased use of EEG with infants and young children (Saby and Marshall, 2012). For decades, research in infants and young children has employed EEG as a methodology to understand the neural processes involved in numerous aspects of early cognitive development (Maguire et al., 2014). Like the adult EEG signal, infant EEG can be decomposed into different frequency bands such as delta (1–3 Hz), theta (3–6 Hz), alpha (6–9 Hz), beta (9–20 Hz), and gamma (>20 Hz), although infant oscillations are generally slower than that of their functional equivalents in adults (Orekhova et al., 1999). Neural activity in different bands has been shown to be of functional significance for the study of a wide range of developmental phenomena including attention (e.g., theta and alpha bands: Xie et al., 2018), face and emotion processing (e.g., alpha band: Batty et al., 2011; Dawson et al., 2012), early language acquisition (Kuhl, 2010), object recognition (Reynolds, 2015), memory (Richards et al., 2010), auditory processing (Telkemeyer et al., 2011), action perception and production, imitation (e.g., alpha band, van Elk et al., 2008; Marshall et al., 2011), and interpersonal synchronization (e.g., alpha and theta bands: Leong et al., 2017; Wass et al., 2018). In particular, early language acquisition research uses brain-to-speech coupling (a measure of how accurately neural oscillatory activity tracks dynamic rhythmic patterns in the speech signal) to study infant-directed speech perception across a number of frequency bands: delta – corresponding to prosodic stress patterns in the speech signal (Leong et al., 2017); theta – representative of the syllabic rate in the English language (Leong et al., 2017; Kalashnikova et al., 2018); and alpha – corresponding to phonemic/onset-rime patterns in speech (Leong et al., 2017). Further, frontal high gamma activity in infants has been associated with the ability to discriminate native from non-native phonetic sounds (55–75 Hz: Peña et al., 2010), and also with inhibitory control and attention shifting skills (31–50 Hz: Benasich et al., 2008). Finally, alpha band power and coherence have been shown to change developmentally in relation to working memory and encoding tasks (Bell and Wolfe, 2007; Köster et al., 2017), while frontal and parietal theta is particularly associated with perceptual binding in learning (Köster et al., 2017).

However, EEG recordings are highly prone to interference by both biological factors (such as electromyogenic activity, the electrical activity produced by voluntary or automatic muscle contractions) and non-biological factors (such as electrical line noise) (Nathan and Contreras-Vidal, 2015). In particular, artifacts induced by motion (such as head motion, jaw motion, or blinking) are a major and common source of EEG distortion. These distortions can result in misinterpretation of underlying neural processes or sources; or to the inaccurate detection and diagnosis of brain disorders (Guerrero-Mosquera et al., 2012). For example, in a concurrent EEG and eye-tracking paradigm, Yuval-Greenberg et al. (2008) showed that the most likely source of the induced gamma-band EEG response – an EEG waveform associated with visual object representation, recognition and attention – were small eye movements made at the onset of each stimulus, rather than a neural response to the stimuli *per se*. Köster (2017) highlighted that a similar, if not worse, problem may exist for infant EEG analyses utilizing activity in the 25–35 Hz gamma range. It has not yet been clarified whether microsaccades (tiny involuntary fixation-related eye-movements) can be measured in infants, whether they generate similar EEG artifacts, and whether the correction methods used for adult EEG signals are applicable to infant data (Keren et al., 2010).

For adult populations, motion artifacts can be avoided or minimized by direct instruction, such as asking participants to only swallow and blink between trials or during other defined periods and asking them to avoid significant head and facial muscle contractions during critical periods of recordings (Reis et al., 2014). However, this strategy is less effective for clinical and pediatric populations whose ability to understand and comply with verbal instruction is greatly reduced. Young infants present a particular challenge in this regard, as they have a high natural tendency for movement, which cannot be constrained by instruction. Indeed, it is widely acknowledged that EEG recordings produced by infants and young children are heavily contaminated by various motion artifacts, including gross motor movements and eye blinks (Bell and Cuevas, 2012).

One common strategy used in infancy paradigms is to reduce motion indirectly through attentional capture – that is, the experimenter monitors the infants’ attentional state through a video feed and only delivers experimental stimuli during attentive periods when the infant is relatively still. However, as exemplified in Supplementary Table S1 (Supplementary Material), our own studies suggest that even when contingently delivered screen-based stimuli are used (including cartoons, real language, and artificial language stimuli), infant movement (i.e., facial, limb or postural movement) is still present throughout 60–70% of the total stimulus presentation time. In more naturalistic paradigms, in which infants are not watching a computer monitor but engaged in social interaction, we might expect that artifacts will be even more prevalent.

### Naturalistic Social Paradigms

The necessity for ecological validity in experimental developmental psychology has been emphasized for decades (Tunnell, 1977; Fabes et al., 2000). It is accepted that the combination of experimental and naturalistic research methods offers a more complete insight into child development (Dahl, 2016). The use of naturalistic methods is more common in social science research than in the neurosciences. However, across a number of neuroscience sub-fields, such as developmental and social neuroscience, the balance is beginning to shift in favor of EEG paradigms with greater ecological validity (for example, see Babiloni et al., 2007; Lindenberger et al., 2009; Dumas et al., 2010, 2011, 2014). Still, a tension exists between the ecological benefits conferred by naturalistic social interaction, and the generation of EEG artifacts from participants’ social behavior (e.g., facial and gesticulatory movements). Observational assessments of behavior in the “real world,” such as in the home or school environment, have higher ecological validity than assessments that occur within structured experiments in laboratory settings, where participants typically perform screen-based tasks that require little or no social interaction. The lack of social interaction is a particular issue for infancy studies. Humans are a social species (Schilbach et al., 2013) and most attention and learning during the crucial early years of life takes place in social settings. For example, social factors influence attention allocation: when a parent pays attention to a particular object during social interaction with their infant, infants’ own attention to the object is increased (Yu and Smith, 2016; Wass et al., 2018). Therefore, when social interaction is excluded from infant experimental paradigms, this can affect the validity and generalizability of early cognition studies.

However, real-world measurements also carry the disadvantage of less (or no) control over key environmental variables that can affect behavior, leading to increased inter-subject variability. As a compromise, naturalistic laboratory settings allow for some controlled task-based variation between participants and facilitate the emergence of more natural behavior whilst at the same time minimizing environmental variation (Noris et al., 2012; Tamis-LeMonda et al., 2017). For example, with infants, such compromises may allow for parental social interaction within a semi-scripted paradigm.

### Common EEG Motion Artifacts

Neural activity at the scalp level is low in amplitude compared to other sources of electrical activity, such as electrical potentials generated by muscle activity and environmental electrical noise (i.e., a low signal-to-noise ratio). Amplification that is applied to the neural signal also amplifies non-neural contaminants, and therefore does not improve neural signal detection. Myogenic EEG artifacts that arise from involuntary movements supporting the physiological functioning of the body, such as heartbeat and respiratory torso movements, can be monitored using a devoted channel, such as an electrocardiogram (ECG), which can significantly improve automated detection and removal strategies (Klados et al., 2011). By contrast, voluntary movement, such as motion generated by the jaw, head, body and limb, are both less easy to monitor through a devoted channel, and less frequently addressed in the literature. For example, in an extensive review of methods for EEG artifact detection and rejection, Islam et al. (2016) found that across 46 publications that were reviewed, over 70% focused solely on automatic movements, with the rest only partially addressing forms of voluntary action. Perhaps best understood are the effects of eye and jaw motions. However, this literature pertains almost exclusively to adults, and very little is known about the nature of motion artifacts in *infant* EEG signals.

A single eye movement can produce a number of artifacts that arise from different mechanisms (e.g., eye rotations and blinks) and differ in their amplitude and spectral properties (Plöchl et al., 2012). Eye movements can introduce systematic biases in both adult (Yuval-Greenberg et al., 2008; Keren et al., 2010) and infant (Köster, 2017) EEG analyses. Jaw movements are another major source of EEG artifacts. In experimental paradigms, jaw motion commonly occurs as a corollary of speech production (Ganushchak et al., 2011). Jaw motion causes significant distortion to EEG signals due to facial myogenic potentials originating from contractions of the frontalis and temporalis muscles when tensing or clenching the jaws (Sweeney et al., 2012). Speech-related articulatory motions are known to reduce the signal-to-noise ratio of neural signals that relate to cognition (Brooker and Donald, 1980). For instance, the myogenic potential generated by the temporalis muscle, used for closing the lower jaw, spreads widely over the scalp frontal/temporal/parietal locations, generating large broadband artifacts in the EEG signals measured over these regions (Brooker and Donald, 1980).

Myogenic artifacts are more problematic for infant than adult measurements since involuntary physiological activity such as heartbeat and blinks are less stereotypical than adults’ and therefore more difficult to identify in the EEG recording (Fujioka et al., 2011). A further complication arises from infants’ tendency to move abruptly and frequently, which creates temporary displacement of channels on the scalp and high-amplitude artifacts (Fujioka et al., 2011; Bell and Cuevas, 2012; Hoehl and Wahl, 2012). Hence, artifacts arising in infant EEG are more challenging to identify and remove using the de-noising procedures normally applicable to adult EEG.

### Current Strategies for Addressing Motion Artifacts

There are two major approaches to addressing the problem of movement-related artifacts in EEG data. Researchers typically (1) exclude artifact-contaminated segments by employing strict rejection procedures/thresholds; or (2) attempt to remove artifacts from data using correction procedures such as independent component analysis (ICA) (Gwin et al., 2010). The first approach (artifact exclusion) is conservative and may entail considerable data loss, especially with infant participants (Fujioka et al., 2011; also see Supplementary Table S1), potentially leading to skewed data and subsequent misinterpretation. Therefore, there is increasing interest in correction procedures that permit the accurate identification and removal of artifacts from EEG data without a significant compromise to the integrity of underlying neural activity.

Several methods have been proposed for the detection and removal of physiologically generated artifacts from the EEG signal. These include the use of linear regression (Klados et al., 2011), Independent Component Analysis (ICA), and blind source separation (BSS) based on Canonical Correlation Analysis (CCA, Vos et al., 2010), to separate the neural signal from interfering electrical signals in the EEG trace. However, none of these methods is able to completely remove motion artifacts from the EEG signal and may even remove some genuine neural activity of interest. As noted by Islam et al. (2016), current artifact detection-removal methods are sub-optimal because these methods typically only address a single artifact class and necessitate dedicated reference channels, and moreover, frequently result in overcorrection. For example, a common approach to addressing some classes of stereotypical artifacts (such as eye blinks) is to include an observed reference channel that independently measures the artifact signal (i.e., EOG for eye muscles, ECG for heartbeat). Next, linear regression or ICA may be employed to estimate the similarities between the EEG signal and the reference signal, permitting removal of the artifact estimate from the EEG signal (Klados et al., 2011). ICA does not require the presence of a reference signal (although it can improve performance, see Plöchl et al., 2012), and is therefore a widely used approach for artifact removal (Chaumon et al., 2015). While all these approaches can be successful for highly stereotypical artifacts (such as heartbeats and eye-blinks in adults), they fail for less stereotypical artifacts that particularly affect infant EEG as these methods are designed to extract repetitive patterns in the signal over many occurrences of the same type of noise (where each noise occurrence presents with a similar shape and form). Further, the placement of additional reference channels (e.g., under the eyes) may not be well tolerated by pediatric participants.

Two correction approaches that have been successfully applied in infant EEG studies are *Independent Channel Rejection* (He et al., 2007) and *Artifact Blocking* (Fujioka et al., 2011). Both methods only eliminate data in trials and/or channels where there is an artifact (defined by amplitude displacement above a certain absolute threshold), without removing the whole trial or channel. Thus, these methods ameliorate the problem of data loss due to artifact rejection, and have been used in a number of research studies as a pre-processing data cleaning strategy (i.e., Corrigall and Trainor, 2014; Folland et al., 2014; Slugocki and Trainor, 2014; Trainor et al., 2014; Agyei et al., 2016). However, these strategies still rely on the successful classification of portions of the EEG signal as artifactual, which is itself a non-trivial task. Classification often relies either on some form of automated pattern-recognition (i.e., machine-learning classifiers) or a combination of automated and manual identification (i.e., ICA where components are rejected by eye). EEG pattern-recognition is challenging due to the large amount of natural variation in the signal, which is exacerbated further by the presence of sporadic artifactual activity, hindering accurate classification.

Newer machine learning approaches have begun to be employed for automatic classification of artifactual and non-artifactual segments of EEG signals. These methods are particularly applicable to motion-related distortions which are less stereotypical. For example, O’Regan et al. (2010) trained a classifier to segregate different types of artifactual neural EEG signals. In their study, 19 adult participants were instructed to perform 32 types of head actions that had previously been related to distortions in ambulatory EEG, including head shaking, rolling, nodding, jaw clenching, lowering and raising of eye-brows. A classifier using linear discriminant analysis was trained from a random selection of 20% of the data and resulted in up to 76.49% accuracy in distinguishing head-motion contaminated EEG. Similarly, Lawhern et al. (2012) investigated methods for automatic detection and classification of EEG artifacts generated by different types of jaw and eye movements. An autoregressive model using a Maximum Likelihood Estimator was used to estimate features for a support vector machine classifier. The procedure was successful in differentiating between broad classes of artifacts (i.e., jaw and eye); but it tended to group together more specific artifacts from a common source. However, the error rate of falsely classifying epochs with no artifacts was low (the reported average accuracy for 5 out of 7 participants was over 96%, and over 81% for the remaining 2). Therefore, newer machine learning approaches may, in future, have strong utility for the detection of more complex classes of motion artifacts. It is anticipated that the data from the current study could be used, in future, to inform the development of such new tools for artifact identification in infant EEG signals.

### Pilot Study to Assess Common Infant Movement Types and Their Prevalence in a Naturalistic Task

A pilot study was conducted to identify the most prevalent infant motions in a naturalistic play setting where infant participants interacted with toys in a social or non-social context. We were interested in identifying motion patterns elicited during social interaction, as naturalistic developmental paradigms often include, or at least permit social interaction. Hence, we identified the most common infant motions produced during naturalistic object-oriented play and also investigated how these differed between social and non-social experimental conditions. The full inventory of infant movements analyzed included Talking, Chewing, Whining, Side-to-Side neck movements, Up and Down neck movements, Small Hand, Small Foot, Large Arm and Large Leg movements. The pilot study is fully described in Supplementary Material section “Pilot Study 1.” Our results revealed that across both play conditions, infant motion was present over 95% of the time, which represents near-continuous contamination of the EEG signal. We further noted that the most commonly occurring types of infant motion were small hand and large arm movements, which is unsurprising given that the paradigm involved object-oriented play. When further considering the types of motions that occurred as a function of play condition (social or non-social), we found that an increase in motion frequency was observed during non-social relative to social play for chewing and nodding movements, and no difference was observed for limb (arm and leg) movements and talking/whining. This pattern suggests that when actively engaged in play with their parents, infants were less distracted and therefore showed a lower tendency to move. Supplementary Material section “Results” presents a detailed account of the full set of results from the pilot study.

### Study Aims

The high prevalence of movement observed during the pilot study motivated a more detailed study seeking to identify the spectral and topographical effects of movement on the infant EEG signal. As the removal of artifacts from EEG data is restricted by current methodological limitations, and clinical and infant populations are unable to comply with directions to reduce movement to lessen distortion of the signal, there is a clear need for research to report how these artifacts distort EEG data. Accordingly, the major aim of this work is to investigate the individual topological and spectral features of commonly occurring motion-related EEG artifacts in infants, as compared to resting state EEG measurements.

## Methods

### Participants

Fourteen infants participated in the study. There were 6 boys and 8 girls in the group, with an average age of 338.85 days (SD = 59.59). Two infants produced insufficient resting state data due to fussiness, and so were excluded from the analysis. The remaining 12 infants comprised 5 boys and 7 girls, with an average age of 325.5 days (SD = 51.77). All mothers reported no neurological problems and normal hearing and vision for their infants. Participants also took part in other separate experiments on the same day as the current study, but these data are unrelated to the current study and will not be reported. This study was approved by the Cambridge Psychology Research Ethics Committee, and parents provided written informed consent on behalf of their children.

### Materials

For the duration of this experiment, the infants saw a series of brief age-appropriate videos. The videos included familiar nursery rhymes (such as “Twinkle Twinkle Little Star”) that were sung by a female adult, interspersed with different (static) cartoon pictures. All infants saw the same set of videos, presented in a counterbalanced order. The videos lasted up to 22.77 min in total.

### Protocol

**FIGURE 1:**
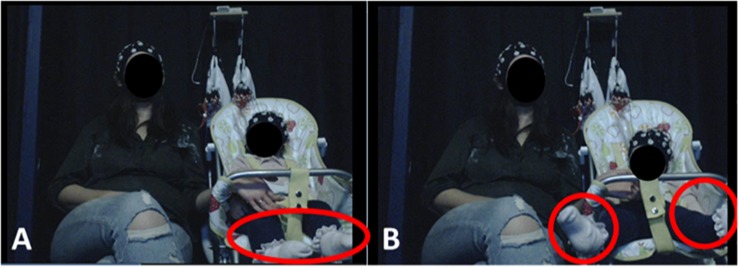
Experimental Setup. Infants were seated on a highchair next to their mothers. A camera, placed in a central location in front of participants, recorded infants’ behavior and motions. **(A)** (left) illustrates resting state behavior, when the infant showed no visible motion. **(B)** (right) illustrates leg movement by the infant. Written informed consent was obtained from the parents for the publication of these images.

Here, infants’ spontaneous motions during passive video viewing were analyzed. As shown in Figure 1, infants passively watched videos while seated in a high chair, with their mothers seated adjacent to them. A passive (video viewing) task was used in order to allow us to better assess the individual contribution of each motion type. As noted in section “Pilot Study to Assess Common Infant Movement Types and Their Prevalence in a Naturalistic Task” and detailed in Supplementary Material section 2.2, during object-oriented play, motions typically co-occurred because infants were actively exploring the toy objects (e.g., infants picked up the toy with their hands whilst bending their necks downward to inspect it). In the recorded EEG signal, the effects of these co-occurring motions would mix and overlap spectrally and topographically, making it very difficult to isolate the individual effects. In a passive paradigm, infants were more likely to make only one type of motion at a time, providing unambiguous exemplars for analysis^1^. The passive design also had the added advantage of optimizing the comparability of motion-related EEG with infant “resting state” EEG. Infant resting state EEG is typically recorded whilst infants quietly watch a screen with some non-arousing video presentation (Bell and Cuevas, 2012). This protocol minimizes frequent eye and motor movements, although some isolated movement by infants always occurs. Here, we capitalized on these isolated infant motions in order to collect both motion-related EEG and resting state EEG *within the same recording*.

#### EEG Acquisition

A 32-channel BIOPAC Mobita mobile amplifier was used with an Easycap electrode system. Electrodes were placed according to the 10–20 international system for electrode placement (see Figure 3D). Data were acquired using Acqknowledge 5.0 software, at a 500 Hz sampling rate. The ground electrode was affixed to the back of the neck as this location is the least invasive for infants.

The 12 infants included in the final analysis produced an average of 325.5 s (SD = 51.8 s, range 274–448 s) of raw continuous EEG recording, which included periods of rest and of spontaneous motion. The raw EEG recording was then segmented to chunks containing only either rest, or a single motion, as operationally defined in later sections.

#### Video Recordings

A Logitech High Definition Professional Web-camera recorded infants’ behavior (at 30 frames per second) throughout the session. Afterward, each video was manually screened frame-by-frame and coded to ascertain the start and end times of each motion type of interest, and of the resting state periods.

#### Video Coding

The motion and rest timings were manually extracted from each video by video coding. Three trained video-coders noted the onset and offset time of each motion or rest period by looking through the recorded video frame by frame.

#### Resting State

Resting state periods were strictly defined as periods during which the infant exhibited fixated gaze with no visible facial or bodily motion and maintained this state for at least half a second. Periods with any visible motion were excluded. On average, infants produced 49.2 s of resting data (SD = 24.4) prior to pre-processing, and 38.3 s (SD = 14.7) of clean resting data after pre-processing.

#### Motion Artifacts

Five types of *infant* motions were selected for the analysis. These included: Jaw movements (e.g., talking/babbling and chewing), and Limb Movements (LMs) of the Hand, Arm, Foot and Leg. Only motions lasting for longer than 250 ms were included for the analysis. An additional inclusion criterion was that only one motion should be present at any time – periods containing overlapping motions were excluded from the analysis. Identical to the resting state data, during motion, infants’ gaze was fixed and no eye-movements other than blinks were present. Infants produced an average of 22.2 s of data per motion type (SD = 19.6 s) prior to data-cleaning, and an average of 18.8 s (SD = 17.9 s) of pre-processed data were included in the final analysis. A detailed operational definition of each one of these motions is given in Supplementary Material sections “Facial Motion Descriptives,” “Body Movement Descriptives,” and “Head Movement Descriptives.” We focused on these motions because they were the most prevalent types of motions made by infants in the pilot study. Statistical stratification was performed to assess the effect of data duration differences on the main reported results (described in Supplementary Material section “Statistical Stratification to Assess for Effects of Data Duration Differences Across Conditions”).

#### Video-EEG Synchronization

Video recordings were synchronized to the EEG signal by sending triggers via a radio frequency transmitter which marked the EEG trace and produced a light signal that was visible on the video recording. Synchronization was performed manually by recording the exact frame at which the onset of the synchronization light signal occurred. Thus, the synchronization accuracy was limited to the temporal resolution of the video frame rate, which was 30 frames per second (33 ms).

### EEG Acquisition and Analysis

#### EEG Pre-processing

Noisy channels with raw amplitude fluctuations above 100 μV above the rest of the channels for over 25% of the recording session were rejected. Table 1 shows the number and location of rejected channels for each infant. Next, the data were re-referenced to the average of the remaining channels. EEG segments containing each type of motion were concatenated, creating separate continuous datasets for each motion type, and for the resting state. These concatenated data were then visually inspected for eye-blinks and high amplitude fluctuations, which were removed unless directly arising from the modeled action.

**TABLE 1:** EEG channels rejected for each infant and movement type.

	**Rejected channels**					
**Infant ID**	**RS**	**Jaw**	**Hand**	**Arm**	**Foot**	**Leg**
1	–	–	–	–	–	–
2	–	–	–	–	–	–
3	CP5	–	–	CP5	–	CP5
4	–	–	–	–	–	–
5	–	–	–	–	–	–
6	T7, TP9	T7, TP9	–	TP9, P7	–	TP9
7	TP9	–	TP9	TP9	–	TP9
8	–	–	–	–	–	–
9	–	–	–	–	–	–
10	C3, CP2	–	C3, CP2	–	–	–
11	–	–	–	Cz, TP9	TP9	Cz
12	T7	–	–	–	–	T7

*RS, resting state.*

#### EEG Power Analysis

To describe the topographical distribution of power by frequency for each condition (rest or single motion type), raw power scores were transformed into *z*-scores to permit averaging across individual infants in a standardized manner. First, each continuous dataset per condition and infant was divided into non-overlapping 1-second-long epochs. A Fast Fourier Transform (FFT) was performed for each epoch, yielding 3-dimensional estimates of spectral power (channel × frequency bin × epoch) for each infant and condition. Next, for each frequency bin, the sample mean of all epochs and channels was subtracted from the sample mean for each channel (averaged over epochs). This differenced data were then divided by the sample standard deviation of all epochs and all channels per frequency bin to derive the normalized power spectra z-scores, as described in eq. (1) below:

$$z\,\_\,p\,o\,w_{\left(c,f\right)}^{\left(i\,n\,f\,a\,n\,t,c\,o\,n\,d\right)}$$

$$=\frac{m\,e\,a\,n_{e}\,\left(p\,o\,w_{\left(c,f\right)}^{\left(i\,n\,f\,a\,n\,t,c\,o\,n\,d\right)}\right)-m\,e\,a\,n_{e}\,\left(m\,e\,a\,n_{c}\,\left(p\,o\,w_{\left(f\right)}^{\left(i\,n\,f\,a\,n\,t,c\,o\,n\,d\right)}\right)\right)}{s\,t\,d\,e\,v_{e}\,\left(m\,e\,a\,n_{c}\,\left(p\,o\,w_{\left(f\right)}^{\left(i\,n\,f\,a\,n\,t,c\,o\,n\,d\right)}\right)\right)}$$

(1) where c = channel; f = frequency bin; e = epoch

For each condition, the standardized power spectra for each channel were then averaged across all infants, according to eq. (2).

(2) $$m\,e\,a\,n\,\_\,z\,\_\,p\,o\,w_{\left(c,f\right)}^{\left(c\,o\,n\,d\right)}=\frac{\sum_{i\,n\,f\,a\,n\,t}z\,\_\,p\,o\,w_{\left(c,f\right)}^{\left(c\,o\,n\,d\right)}}{n_{i\,n\,f\,a\,n\,t}}$$

For clarity of reporting, spectral power in the following scalp topographical plots is reported as averaged over pre-defined frequency bands. As infant oscillations are generally slower than their functional equivalents in adults (Orekhova et al., 1999), the standard EEG frequency bands were downward-adjusted accordingly: delta (1–3 Hz), theta (3–6 Hz), alpha (6–9 Hz), low beta (9–13 Hz), and high beta (13–20 Hz) (Leong et al., 2017).

#### Cluster-Based Permutation Test of Motion-Related Power Changes

Statistical comparison of spectral power differences between motion and resting state data was conducted using the Matlab-based toolbox, Fieldtrip (Oostenveld et al., 2011). First, frequency decomposition of the pre-processed data was performed using a multi-taper FFT based on discrete prolate spheroidal sequences (DPSS) and 2 Hz smoothing frequency, for the frequency range of interest 0.1–20 Hz (*ft_freqanalysis*). The derived power spectra (uV^2^/Hz) during the resting state and each motion type were thus calculated separately for each infant, and then a grand-average was calculated across infants using *ft_freqgrandaverage*. Next, statistically significant differences in power between each motion type and the resting state were assessed at the group level by conducting a within-subject non-parametric cluster-permutation test. We corrected for multiple comparisons using Monte-Carlo estimates of the two-tailed significance probabilities (alpha = 0.05) from the permutation distribution based on 10,000 permutation cycles, using Fieldtrip’s function *ft_freqstatistics*. This procedure identified clusters of neighboring sensors where the EEG power differed significantly between a specific type of motion and the resting state data (in either direction), and is particularly suitable for use with non-parametric datasets (Maris and Oostenveld, 2007). Clusters were defined with a minimum of three sensors per cluster (with one unit distance between neighboring sensors, and yielding an average of 6–7 neighbors per sensor).

## Results

### Motion Types and (Isolated) Prevalence

The total duration of each type of motion (occurring in isolation) for each infant is shown in Table 2. All 12 infants whose data were analyzed produced motion in at least two out of five motion categories. As not all the infants spontaneously produced all types of motion, the number of participants analyzed for each motion type varied between 4 and 10 (see Table 2).

**TABLE 2:** Total duration (in seconds) of clean pre-processed isolated motion and resting state EEG contributed for the final analysis by each infant.

	**Total duration contributed (s)**					
**Infant ID**	**RS**	**Jaw**	**Hand**	**Arm**	**Foot**	**Leg**
1	15	8	7	5	4	19
2	46	–	14	11	8	27
3	53	8	–	10	–	57
4	33	14	–	6	–	–
5	10	18	12	10	4	18
6	36	10	27	11	–	21
7	34	–	25	28	–	14
8	36	4	–	–	–	4
9	44	–	41	9	–	5
10	59	–	27	–	–	–
11	54	–	36	27	93	51
12	40	–	–	5	–	6
Average(SD)	38.3(14.7)	10.3(5)	23.6(11.9)	12.2(8.4)	27.3(43.9)	22.2(18.4)

Note that there was a difference in the prevalence of the same motions reported in the behavioral pilot study and here. This is because the behavioral pilot (see Supplementary Figure S3 in Supplementary Material section “Results”) considered all occurrences of motion present during the task (including co-occurring motion), whereas here we only report isolated motion.

### Scalp Topographies by Frequency Band

#### Resting State

**FIGURE 2:**
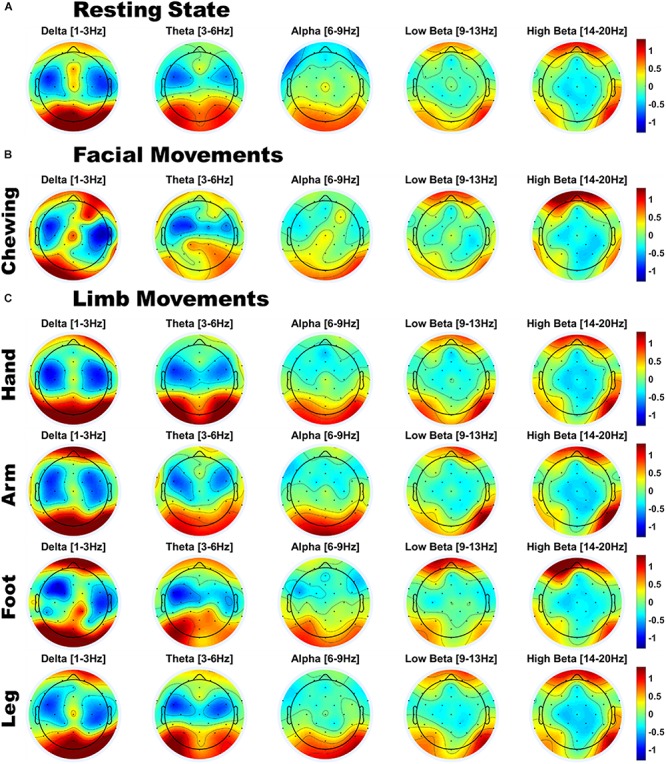
Scalp topographies of infant EEG power for **(A)** Resting state; **(B)** Facial movements; and **(C)** Limb movements; Power is *z*-normalized power [uV^2^/Hz], averaged over infants. Red indicates a region of above-average power, and blue indicates a region of below-average power.

As shown in Figure 2A, infants’ resting state scalp topology was characterized by high power over posterior regions in delta and theta bands, and high alpha power over centro-parietal regions. Additionally, beta power was higher over bilateral orbitofrontal regions, while it was relatively lower over bilateral temporal regions, which could reflect the presence of oculomotor activity (such as microsaccades). Individual plots for each infant’s resting state scalp topologies are presented in Supplementary Figure S4A (Supplementary Material section “Individual Infants’ Scalp Topographies During Resting State and Motion”).

#### Movement

Compared to resting state EEG, the scalp topology of infants’ movement EEG also showed a broadly similar pattern of high delta/theta power over posterior regions and high beta power over orbitofrontal regions (see Figures 2B,C). However, visual inspection also indicated variations in scalp topography by movement class. To assess whether there were significant patterns of spectral and topographical difference in the power spectra of motion relative to the resting state data, a cluster permutation analysis was applied (see section “EEG Acquisition and Analysis”). Individual plots for each infant’s motion-related scalp topologies are presented separately for each motion type in Supplementary Figures S4B–F (Supplementary Material section “Individual Infants’ Scalp Topographies During Resting State and Motion”). Here, the group average topologies are presented.

### Motion-Related Differences in Spectral Power and Topography

**FIGURE 3:**
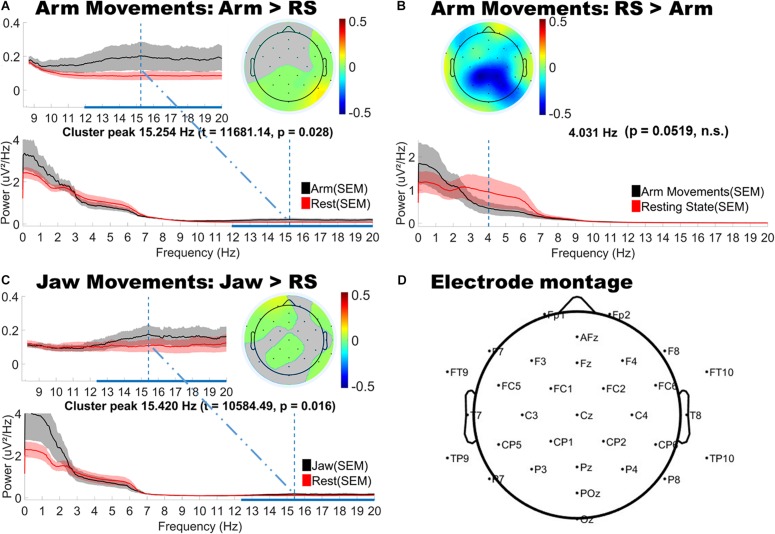
Topographical and spectral differences in infant EEG power for **(A,B)** Arm and **(C)** Jaw Movements relative to resting state. The line plots below show the power spectra for motion (black line) and resting state (red line). Panel **D** shows a map of the locations of the electrodes on the head. **A,B** and **C**: The horizontal blue line on the *x*-axis indicates the frequency range over which significant differences in power were observed. The vertical blue line shows the peak difference in frequency and the headplots above this show the scalp topography of the cluster at the peak difference in frequency. Gray areas in the headplots **(A,C)** show non-significant difference. The color bars indicate differenced power.

As shown in Figure 3, jaw movements and upper limb movements (arm) did indeed produce significantly *increased* low and high beta (12–20 Hz) power, peaking at around 15 Hz for both motion types and most strongly observed at peripheral sites. Jaw movements generally produced anteriorly located increases in beta power, particularly over frontal and fronto-temporal regions bilaterally. Smaller central increases in beta power were also observed. By contrast, arm movements mainly generated posterior increases in beta power, strongest over posterior parietal and occipital sites. Lower limb movements (foot and leg) and hand movements produced no significant changes as compared to the infants’ resting state topography.

The cluster analysis also revealed trends toward *decreased* power in the theta and alpha bands, however, these did not reach statistical significance (*p* = 0.0519). These trends in theta and alpha decreases were consistently observed over central topographies for all motion types (see Figure 3B for example).

## Discussion

Electroencephalography recordings are highly prone to distortion by motion-related artifacts, which can result in the misinterpretation of underlying neural processes, or even the inaccurate detection and diagnosis of brain disorders (Guerrero-Mosquera et al., 2012). Young infants present a particular challenge as they have a high natural tendency for movement, which cannot be constrained by instruction. This work represents the first systematic assessment of the effects of naturalistic (social) infant motion on the recorded EEG signal. It is intended that this work will build toward a more comprehensive database or “Artifact Library” which could later serve as a common resource for EEG researchers in social and developmental neuroscience.

In a behavioral pilot study, we assessed the prevalence of motion in adult-infant dyads during social and non-social naturalistic play paradigms. We observed that motion occurred >95% of the time, for both infants and adults, and in both types of social play settings. For such datasets, it would not be feasible to adopt a simple approach of rejecting (excluding) all motion-contaminated data, as this would entail losing an unacceptably high proportion of data. However, before artifact removal methods (such as ICA or CCA) can be effectively applied to the EEG signal, it is first necessary to understand the exact distortion that these motions would produce. Accordingly, the current study attempted to document the topographical and spectral properties of each of the most prevalent (frequently occurring) types of motion that were observed in the behavioral pilot. Here, infants’ motions, produced one at a time during a passive video-viewing paradigm, were analyzed. The spectral properties of these motion-contaminated signals were then contrasted against a resting state baseline.

In general, the infants’ motions generated only a few significant deviations from the resting state power spectrum. Upper limb movements (arm) and jaw movements (e.g., chewing) produced stronger and more widespread artifacts than hand movements and lower limb movements (foot and leg). Further, infants’ arm and jaw movement artifacts were both characterized by *increased* beta power which was most evident at peripheral sites. Jaw movements generated mainly anteriorly located increases in beta power over frontal and fronto-central sites whereas arm movements produced strong posterior increases in beta power over parietal and occipital regions. By contrast, lower limb and hand movements produced no discernible changes in infants’ spectral power.

One potential explanation for the apparent limited effects of motion contamination in infants’ EEG data could be that insufficient data were collected from infants to reveal true differences. Data from only 12 infants were included in the final analysis, with an average of 38.3 s (14.7) of rest and of 18.8 s (SD = 17.9 s) of single motion segments per infant. However, this explanation is unlikely as the main results were visually similar to the effects of motion described in our supplementary study where the number of trials produced by the infant was comparable to the data quantity produced in a controlled laboratory experiment with an adult (e.g., the mother of the infant produced a 450 s of resting state data compared to 328 s produced by the infant; and the infant also had a higher number of recorded instances for all motion types than his mother). An alternative explanation could be a bias in data quantity in favor of the resting state condition. However, an additional cluster-based permutation analysis on a stratified subsample of the data (reported in Supplementary Material section “Statistical Stratification to Assess for Effects of Data Duration Differences Across Conditions”) following the original protocol showed that Arm-related effects were virtually identical. Although the Jaw-related effects did not reach significance in the stratified subsample (due to loss of power), similar spectral trends in the data were observed. These supplementary analyses confirm that data duration differences did not introduce systematic biases into the results. Furthermore, it should be noted that infants’ resting state data differed qualitatively from resting state data that is typically collected from adults. Since we could not instruct infants to produce a state of rest, their resting state data was collected incidentally (e.g., whilst watching a video) and periods of non-motion were identified through video coding. This protocol of recording continuous EEG and selecting relevant segments offline is frequently used in infant research (e.g., Orekhova et al., 2014). Still, it is possible that this procedure inadvertently included some tonic muscle activity that was not visible on video, leading to an underestimation of the true extent of the effects of motion on the infant’s EEG signal.

Although not statistically significant, we observed trends toward decreased theta and alpha power at central sites associated with all motion types. Alpha or “mu rhythm” suppression has been well-documented in infants and young children in relation to both action production and action observation (Liao et al., 2015). For example, Marshall et al. (2011) reported suppression effects in the 6–9 Hz range, broadly distributed over the scalp, when 14-month old infants were engaged in action observation in a social context. This raises the question of whether the movement-related suppression effects observed here are truly “artifactual”, since they could also reflect the cognitive or social processes that underpin infants’ action generation, and therefore are more in the realm of confounding neural processes as opposed to truly artifactual signals that originate from peripheral muscle or electrode movement. According to this view, only unintentional or involuntary movement produces truly artifactual effects. However, to definitively separate these effects would require concurrent measures of intentionality and cognitive processing, along with active and passive manipulations of participant motion, which are beyond the scope of the current study.

### Implications for EEG Research Using Naturalistic Paradigms

The growth of naturalistic EEG paradigms reflects the view that movement is a natural neural state (Makeig et al., 2009) and that cognitive processes themselves are embodied (Gramann et al., 2011). The brain maintains representations of its internal (proprioception) and external (motor behavior and audio-visual scene) environment – and these representations are constantly, dynamically updated through action. To study cognition in this holistic and action-oriented way, new technologies and imaging methods are required, such as mobile sensors that can synchronously image the brain (i.e., wireless EEG) and the body (i.e., motion capture) and perform online registration of the two modalities. One such system is the sensor technology for mobile brain/body imaging (MoBI) which has shown promising results for studying changes in the EEG power spectrum in relation to participants’ gait during locomotion (Gramann et al., 2011). The MoBI system has also been used to localize independent components in relation to motions such as head-turning, pointing, and walking in a 3D virtual reality orientation task (Makeig et al., 2009). However, when such sophisticated methods for tracking and removing EEG artifacts are not available, precautions should be taken when analyzing data from naturalistic paradigms, as discussed next.

Our results indicated that for infants, one likely effect of motion was reduced EEG power over central sites in the theta and alpha bands. Accordingly, if EEG researchers are investigating phenomena where infant alpha band effects are predicted (e.g., mu de-synchronization or emotion-related frontal alpha asymmetry), care must be taken to avoid including confounding jaw or limb motions, which can independently create changes in alpha band power across frontal and central sites. Additionally, EEG researchers studying speech perception or brain-to-speech synchronization in the beta bands might be cautious when interpreting results if jaw or arm motion is present during stimulus presentation. For example, jaw motions may provide a confounding factor when infants suck on a teething toy whilst watching screen-based experimental stimuli. It is important that experimenters check, and report (e.g., by video coding) the relative occurrence of these movement artifacts in infant participants across experimental conditions and groups, to verify that any reported results cannot be explained by differences in patterns of movement. Further, we also found that infants’ peripheral EEG channels were particularly vulnerable to motion-related power increases, and therefore recommend particular caution (or exclusion) when analyzing these channels.

### Implications for EEG Research Using ERP Paradigms

Motion artifacts present a different set of challenges for research employing the use of evoked-response potentials (ERPs, sometimes also referred to as event-related potentials). ERPs measure changes in the electrical potential of the EEG trace in response to a discrete event in time, in some or all electrodes. ERPs are thus time-locked responses averaged over a number of repetitions (trials) of the same event. The measurement of ERPs is perhaps the most widely used EEG method in the infant literature. ERPs have been used to study face perception (e.g., de Haan and Nelson, 1999; Halit et al., 2004); visual recognition memory (deRegnier et al., 1997); the maturation of auditory perception (e.g., Kushnerenko et al., 2002); auditory recognition memory (e.g., deRegnier et al., 2000, 2002); word recognition and language processing of phrases based on intonation (Mannel and Friederici, 2009; Mannel et al., 2013). The negative central component (NC), which is the developmentally earliest described endogenous ERP component, is present in newborn infants (Nelson, 1996) and has been associated with aspects of attention (e.g., Richards, 2003), memory (e.g., Nelson et al., 1998; Pascalis et al., 1998; Lukowski et al., 2005), and face recognition (e.g., de Haan and Nelson, 1997). By far the most widely used and reported ERP component in the developmental literature is the mismatch negativity (MMN), a negative deflection which arises as a pre-attentive response (Kraus and Naatanen, 1995) to an oddball stimulus embedded within a sequence of standard stimuli (the typical probability of the oddball stimulus is about 10–20%). The MMN is implicated in the discrimination of phonemes (e.g., Winkler et al., 1999) and native vs. non-native stress patterns (e.g., Weber et al., 2004, 2005), and also in the ability to perceive complex statistical regularities such as embedding (nesting rules between stimuli) (e.g., Winkler et al., 2018).

Unless motion is time-locked to the neural phenomenon of interest (e.g., saccades to visual stimulus onset), the effect of motion will vary from one trial to the next, introducing a random (rather than systematic) bias. However, sufficient repetitions will decrease the effects of non-time-locked motion on the ERP signal. Nonetheless, a high prevalence of motion in an ERP paradigm may still reduce the signal-to-noise ratio (SNR) of the effect of interest, when compared to motion-free data of a similar quantity. A reduced SNR has two main effects on the ERP: (1) it reduces the amplitude of the ERP and (2) reduces the precision with which the latency of EEG components can be estimated. These effects also depend on the exact topography of the ERP component of interest. Future studies are needed to investigate these effects in more detail.

The current study may be of particular relevance to ERP research in situations where the amplitude or latency of the neural response is related to neuro-oscillatory processes in one or more frequency bands. Such event-related oscillations (EROs; Csibra and Johnson, 2007), also referred to as event-related synchronisation (ERS), are frequency-band specific bursts in EEG activity that are loosely time-locked to a specific event (for example, the gamma bursts described in the Introduction section “Motion in EEG Measurements”). EROs represent a sustained response to stimulation and have been used to study rapid auditory processing and acoustic change detection (e.g., theta: Musacchia et al., 2015; theta and gamma: Musacchia et al., 2017; Cantiani et al., 2019); infant-directed speech processing (delta and theta: Zhang et al., 2011); discrimination of native and non-native syllable contrasts (delta, theta and gamma: Ortiz-Mantilla et al., 2013); and perceptual binding and object permanence (broadband gamma: Csibra et al., 2000) in young infants. Accordingly, motion which affects spectral power in the frequency band(s) of interest would confound the detection and accurate measurement of the ERO/ERS event. Still, it is worth noting that an ERO/ERS (as well as any other ERP) study design would be less affected by random non-time-locked motion-related artifacts than designs using continuous data analysis, for example when studying naturalistic social interactions, sustained attention, connectivity, or resting-state default-mode networks, to name a few.

Another interdependency between neural oscillatory processing and ERPs is through variations in attentional state. Different attentional processes (alerting, orienting, executive control, sustained attention) are strongly correlated to the neural activity in the theta and alpha bands (in infants and adults: Fan et al., 2007; Xie et al., 2017), but also to beta and gamma band activity (in adults: Fan et al., 2007). The amplitude and latency of attention-related ERP components is modulated by attentional state (e.g., Fu et al., 2005; for review, see Luck et al., 2000), and also by underlying endogenous oscillatory fluctuations in theta and alpha bands (e.g., Buzsaki, 2006; Vanderperren et al., 2008; Harris et al., 2018). Therefore, one may envisage a scenario in which frontal/central alpha desynchronization, which is related to sustained attention in infants between 10 and 12 months (Xie et al., 2017), is potentially affected by infant hand motions that decrease central theta/alpha power and become more frequent during periods of infant inattention. This in turn may produce systematic artifactual differences in the measured ERPs to stimuli presented within a sustained attention paradigm.

It is important to note that based on the current study alone, it is not possible to estimate how the effect sizes of measurements (ERP or time/frequency-based) in a given paradigm compare to the effect sizes of these artifacts. Therefore, EEG researchers may need to assess whether the between-condition effects in their experiment are significantly affected by motion artifacts, especially in cases where there is a difference in movement between conditions, or where the phenomenon under investigation may be biased by motion.

### Implications for ICA Artifact Removal

Independent component analysis is one of the most frequently used techniques for removing motion artifacts from adult EEG data. However, its use in infant EEG is still limited as the spectro-temporal signatures of motion in infant EEG are not as well described as adults’. To assess whether the findings of the current study may be used to guide and improve ICA correction of infant EEG data, we conducted a case study using infant Arm movement data. Arm movements were selected since this class of motion generated the most widespread artifactual effects. The full details of this supplementary analysis are provided in Supplementary Material section “ICA Analysis.” Briefly, independent components (ICs) were rejected in two stages: (1) ICs clearly pertaining to eye movements (blinks and saccades) were removed from both Resting State (RS) and Arm movement data, and (2) ICs specifically related to Arm movement - whose identification was guided by the spectral difference maps produced in the main “Results” section – were only removed from Arm movement segments. Next, we computed the spectral topographic difference maps of the “cleaned” Arm movement data with respect to the RS data, using the same statistical procedure as in the main “Results” section. We found that guided-ICA was indeed successful in removing infants’ Arm movement-related artifacts. Although both positive and negative differences clusters were still present, none of them reached significance. Still, it has to be cautioned that even when guided, the ICA procedure was most likely unable to fully separate Arm movements from other similar but non-related neural activity (we noted that Arm movement-related activity was observed to be spread across 3–6 different ICs in individual infants). Thus, some Arm-movement related activity may have remained in ICs that were not removed. In summary, the results presented in this study (specifically Figure 3) may indeed be used to guide the targeted removal of jaw and arm movement-related ICs from the infant EEG signal. This may have some benefit in reducing the impact of excessive motion, but is unlikely to completely eliminate artifactual motion effects.

### Limitations and Future Directions

The major limitation to the current work is that the study was conducted with a small sample size (*N* = 12). This limits the wider generalizability of these findings, as individuals may differ substantially in their motion patterns, and also in the effects of motion on their EEG signals. Further, given the higher variability of infant (as compared to adult) data and lower signal-to-noise ratio, it is possible that significant effects could have been missed, thereby underestimating the effect of motion on the EEG signal. Therefore, given the very limited quantity of the infant data reported here, caution is recommended in interpreting these findings before subsequent replications are conducted.

The second major limitation of this work is the use of a passive (video-watching) as opposed to an active (i.e., play) paradigm to facilitate better isolation of the effects of individual motions (i.e., reduce the co-occurrence of different motion types). As we were concerned that the movements produced by infants in a passive paradigm may differ from movements during an active task, we conducted a supplementary study in which we repeatedly elicited each type of motion from one mother-infant pair, whilst their EEG was recorded. This analysis (fully detailed in Supplementary Material section “Supplementary Pilot Study 2 on Actively-Elicited Motion”) revealed that, similar to what we observed in the current study, the infant’s motions generated only a few significant deviations from his resting state power spectrum, and the effects of upper limb movement were larger than the effects produced by chewing or lower limb movement. Unlike in the current study, *decreases* in alpha power (mainly over fronto-central, central and centro-parietal regions) produced by the upper limb movements did reach significance in the Supplementary study. Significant *increases* in spectral power in peripheral scalp regions were also present when the infant was “modeling” upper limb and chewing movements, similar to the results observed in the current study when those motions occurred spontaneously. In future, a study actively modeling all motions with a larger number of infants is needed to ensure the replicability and generalizability of the current findings. Also, building on this work, future studies are required to explore the impact of multiple co-occurring motion types on the infant EEG signal. However, the current study, though highly limited, is an important first step in this direction.

Finally, it should also be noted that, as infants’ EEG signals were acquired whilst they were watching a movie, the neural activations recorded would also reflect sensory and cognitive processing of the video stimuli, in addition to the motion-related activity of interest. However, given that the resting state recordings were also obtained during the *same* movie stimuli, the subtraction procedure employed here should result in the removal of most common perceptual effects. Nonetheless, it is possible that infants moved more during some parts of the movie than others (e.g., sections that were more interesting or arousing), leading to potential biases in the data. Future studies may consider the use of video stimuli that present uniform stimulation throughout the task.

Despite these limitations, the current work is a necessary first step toward a better understanding of the effects of motion on infant EEG data. Further studies with a larger number of participants, and a wider range of modeled motions (collected across different social interactive scenarios) will be necessary to ascertain the extent to which these effects are generalizable, and to inform the future development of methods for EEG artifact removal.

## Data Availability Statement

The anonymised and non personally-identifiable EEG datasets generated for this study are available on request to the corresponding author.

## Ethics Statement

This study was approved by the Cambridge Psychology Research Ethics Committee, and parents provided written informed consent on behalf of their children.

## Author Contributions

SG and VL designed the study. SG, SL, and MY contributed to data collection and coding. SG, SL, VN, SW, and VL completed the data analysis and contributed in the writing and revisions of the manuscript.

## Conflict of Interest

The authors declare that the research was conducted in the absence of any commercial or financial relationships that could be construed as a potential conflict of interest.

## Footnotes

1 Note that we also ran a second pilot study (described in Supplementary Material section “Supplementary Pilot Study 2 on Actively-Elicited Motion”), where an infant-adult dyad actively modeled the motions observed in the first pilot study using the same toys to elicit the same motions for the infant. In this second pilot study, the infant produced collectively more than 3 h of EEG recording over the course of three separate testing sessions; the adult produced around 20 h of EEG over 7 testing sessions. Similar to the first behavioral-only pilot study, the co-occurrence of motion was very high in this active protocol and we were unable to identify sufficient isolated repetitions for each infant motion. This necessitated the choice of a passive paradigm for the main study.
