# Supplementary material for: Toward the Understanding of Topographical and Spectral Signatures of Infant Movement Artifacts in Naturalistic EEG
Source: Front Neurosci. 2020 Apr 28;14:352. doi: 10.3389/fnins.2020.00352 (PMC7199478; doi:10.3389/fnins.2020.00352)
Supplement: Supplementary file 1 [file Data_Sheet_1.pdf]

SUPPLEMENTARY MATERIALS

1 Infant Movement During Screen-Based Experiments

1.1 Methods

Data from three experiments in which infants of comparable age (8-12 months) had participated were analysed for the presence of motion. In the first experiment, 11 infants (mean age = 347.4 days [SD = 66.3 days], 7M/4F) watched brief cartoons (14 cartoons, length 7-30 seconds), while seated in a high-chair next to their parent. In the second experiment, 24 infants (mean age = 352.1 days [SD = 64.3 days], 11M/13F) were presented with videos (7 in each condition, length 11-30 seconds) of a female singing nursery rhymes. In the third experiment, 14 infants (mean age = 280.0 days [SD = 15.3 days], 7M/7F) were presented with 20-second videos of a female speaking an artificial language. For the last two experiments, infants were seated in their parent's lap. Parents were instructed to provide passive reassurance but not to restrict their infant's movements, whilst avoiding movement themselves. Both video and EEG data were recorded during all experiments, and infants' behaviour was coded off-line to identify periods of attentive watching without overt movements. The coding criteria for attentive looks were as follows:

- Identify continuous (unbroken) looks that are at least 0.5s in length and appear intentional.
- Include only periods of "good on-task behaviour".

Exclusion criteria included:

- Crying/fussing/inattentiveness
- Touching/moving EEG cap
- Engaging in non-task related behaviour (e.g. drinking from sip cup)
- Very large body and limb motions (e.g. banging toy on table, head jerking backwards and forwards, kicking)

For the current analysis, all looking times coded as "good" were summed per infant and subtracted from the total time of task for each experiment. The remaining time was considered to contain motion. The mean and SD of the motion-contaminated recordings in all three experiments are reported in *Table S1*.

1.2 Results

Table S1. Prevalence of infant motion during three different screen-based experimental paradigms, expressed as a percentage of the total stimulus presentation time. Table S1 is based on 3 independent experiments where infants were video-recorded while watching various stimuli (cartoons, real and made-up language). Recordings were subsequently coded for segments free of muscle movements of any kind. Here we report the average (across all infant participants) amount of time while performing the task and moving simultaneously.

**Stimulus**	**Cartoon clips** *N = 11*	**Speech (nursery rhymes)** *N = 24*	**Speech (artificial language)** *N = 14*
Average prevalence of infant movement (% of total stimulus presentation time)	59.2%	70.1%	71.0%
SD	*23.6%*	*18.6%*	*14.9%*

2 Pilot Study 1

2.1 Methods

2.1.1 Participants

Five infants (1M, 4F) took part along with their mothers. Infants were aged 374.80 days on average (SD = 74.42 days). Four of the five mothers were native English speakers, and one was a native Dutch speaker but her infant was exposed daily to English. The mean age for adults was 33.00 years (SD = 3.03 years). All adult participants reported no neurological problems and normal hearing and vision for themselves and their infants. This study was approved by the Cambridge Psychology Research Ethics Committee, and parents provided written informed consent on behalf of their children.

2.1.2 Materials

The set of toys were appropriate for the infants' age and included toys of differing shapes, textures and colours to encourage infants' interest in playing with them. All toys were made from wood, plastic or fabric and were colourful and small enough to be handled easily by infants. Toys which contained moving parts produced no noise. Examples include a colourful wooden caterpillar, a fabric shoe with a Velcro strap, a wooden bead maze and a colourful wooden Winkel toy (see Figure S1).

2.1.3 Protocol

The purpose of this study was to describe and compare the movements produced by the infants while they were playing with objects separately or jointly with their mothers. In the experimental setup, the infant sat in a high chair, with the adult facing him/her across a table. Joint and separate play sessions each lasted approximately ten minutes and were conducted in a counterbalanced order across participants. During both conditions, an experimenter ensured that participants were playing as instructed, and she provided new toys simultaneously to both the adult and the infant as required to sustain their attention and interest. The experimenter explicitly avoided making prolonged social contact with either the infant participant, or their mother.

Separate play (SP) condition. A 40 cm high screen was set up across the table, so that the infant could see the adult, but neither had full visibility of each other's face (this was necessary to ensure that play activity was functionally separate, but the infant did not become distressed). Figure S1a shows an example of the experimental set-up. Infant and adult were each given a toy to play with independently, and the adult was asked to direct her full attention to the toy whilst ignoring the infant. The adults and infants were given the same toy to ensure that their visual and tactile experiences would be similar.

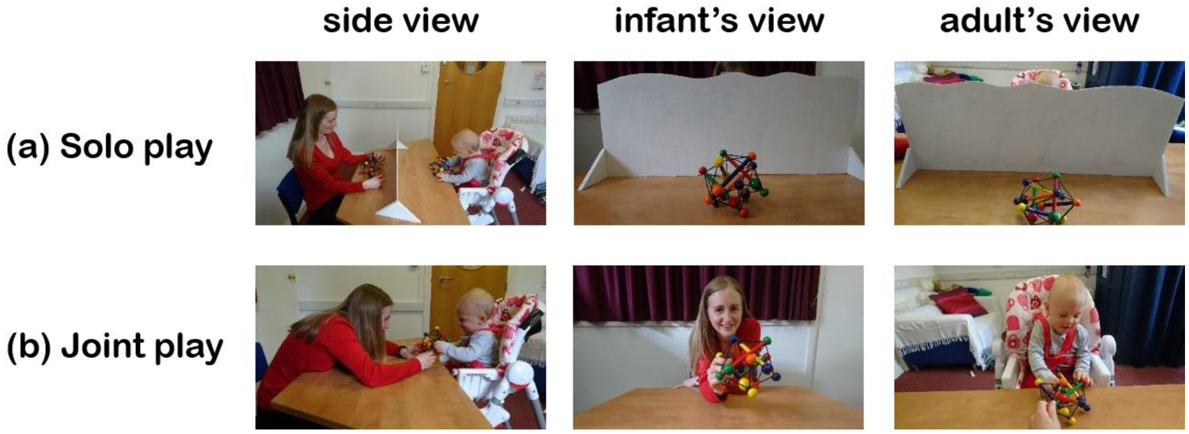

Figure S1. Example of the (a) separate play and (b) joint play experimental set-up showing the (left column) side view (middle column) infant's view; and (right column) adult's view. Written informed consent was obtained from the parents for the publication of these images.

Joint play (JP) condition. For the JP condition, the adult participants were asked to play with their infant using the same toys provided in the SP condition (see Figure S1b). In the JP condition, the adult was requested to actively engage the infant's attention using the toy objects. However, the adult was asked to play *silently* with her infant for two reasons. First, we were primarily interested in facial and body movement-related artifacts (directly related to naturalistic play and social interaction) rather than speaking-related artifacts *per se*. Second, during the SP condition, adults were not speaking and we wanted to keep the acoustic environment across the two conditions similar. In both conditions, therefore, the adults were not speaking. However, infants occasionally made vocalisations in both conditions, which were included in our analysis.

2.1.4 Video recordings

To record the actions of the participants, two Logitech High Definition Professional Web-cameras (30 frames per second) were used, directed at the adult and infant respectively. Afterwards, each video recording was manually coded for the behaviours of interest.

2.1.5 Motion coding

The videos were first reviewed to qualitatively identify different classes of facial and body motions that were produced by the adult and the infant. This analysis was used to devise a coding scheme encompassing 27 different facial and body motions as summarised in Table S2 below and detailed further Sections S2.1.6 - S2.1.8). All infants and adults were scored on all of the items on the coding

scheme although some of them were more characteristic of the infants, while others of the adults.

Table S2. List of facial, body and head movements. Full descriptions of how these were defined are given in Sections S2.1.6 - S2.1.8.

Facial Motion	Body Movement	Head Movement
Talking	Small hand	Left-right nodding
Lip movement	Large arm	Up-down neck movement
Crying	Kicking	Side-side neck movement
Laughing	Small foot movement	Tongue movement
Shrieking	Large leg movement	Arching back
Coughing	Leaning forward	
Sucking	Leaning back	
Whining	Bouncing	
Chewing	Banging	
Jaw movement	Tapping	

Each participant's video was parsed into 5s-long non-overlapping epochs. Within each epoch, the occurrence of each motion was coded as being either present [code = 1] or absent [code = 0]. The total frequency of occurrence for each motion over the entire session was then computed for each participant. Co-occurrences of different motions (e.g. chewing and small hand movement) were frequently observed. From the coded frequency data, comparative statistics were computed to identify (1) commonly occurring motions in each condition, and (2) motions that differed between social and non-social conditions. An example of the coding arising from an infant-mother pair is given in Figure S2.

2.1.6 Facial Motion Descriptives

Talking: This motion comprised sounds such as "baba", "lala", "dada", "coocoo": any generated sounds characteristic of infant babble. The adults were asked not to verbally communicate to the infant.

Lip movement: This motion was most frequently produced through smiling or kissing actions. As the adults were asked not to communicate vocally with the infants, this took the form of a means of communication.

Crying: A noticeable and often continuous cry, signalling frustration or discomfort. This motion was only observed in the infants. Crying might include wailing and whimpering but was unlikely to include tears.

Laughing: This motion comprised chuckling and gurgling, or any sound typical of infantile laughter.

Shrieking: This was a piercing short high-pitched sound or squawk. This was only observed in infants when they were excited or frustrated.
Coughing: This was a regular coughing action.
Sucking: This motion was produced when an infant was sucking on a toy, or possibly their hand.
Whining: This was a long complaining cry or sound. Although often one continuous noise, it may have been broken up into smaller whimpers, often observed when the infant was frustrated or fatigued.
Chewing: This motion was a standard chewing motion and was most frequently observed when the infant was chewing on a toy or their hand.
Jaw movement: This was a relatively animated, noticeable, or extensive movement from the jaw, that is not related to chewing or sucking. Yawning is a good example of this type of motion, and it may also have been demonstrated by an adult producing facial gestures to entertain or attract the attention of the infant.

2.1.7 Body Movement Descriptives

Small hand: This motion comprised finger movements, the twisting of wrists, hand shaking or waving, finger-pointing, and grasping. It encompassed any movement from the wrist, including hand or fingers.
Large arm: This motion comprised full arm extensions in any direction, at any speed, and includes movement from the shoulder and elbow. Reaching forward to grasp a toy was a typical action that induces this motion in an infant and was most commonly produced by the adult when extending their arm to give a toy to the infant.
Kicking: This motion was produced from the legs and was usually demonstrated during moments of excitement or frustration by the infants.
Small foot movement: This motion involved movement from the ankle or toes and is often observed when the infant rotates their foot.
Large leg movement: This comprised any action performed by the legs produced from the hip, regardless of the magnitude of the motion or the speed.
Leaning forward: This occurs when the participant leaned forward in their chair and was frequently observed when the mother reaches forward to hold her baby's hand or to provide comfort.
Leaning back: This motion was produced when the participant leaned back in their chair. It was most frequently observed in adults after leaning forward to comfort or play with their child.
Bouncing: This motion was produced when the participant bounced up and down, or forwards and backwards slightly rising in the seat. This was only observed in infants.
Banging: This was a banging motion produced from the arms and hands, and was observed when participants banged their hands, or toys, on the table.
Tapping: This was a tapping motion produced by the hands.

2.1.8 Head Movement Descriptives

Left to right nodding: This motion was produced when the participant moves their head in a relatively fast paced nodding action from side to side.

Up-down head movements: This motion was produced when the participant moved their head either upwards or downwards slowly, and then maintained it in that position for a short period of time.
Side to side head movements: This motion was produced when the participant moves their head slowly to the left or right and then holds it in either position for a short period of time.
Front to back nodding: This motion was produced when the participant moves their head in a relatively fast paced nodding action up and down.
Tongue movement: This motion was produced when the participant visually protruded the tongue outside the mouth, such as the licking their lips or in a playful gesture such as blowing raspberries and was usually observed when adults are attempting to entertain their infant.
Arching back: This motion involved the participant arching back and pushing back hard into their seat. The neck and head were usually fully extended backwards, and this motion was most frequently observed in infants during times of frustration.

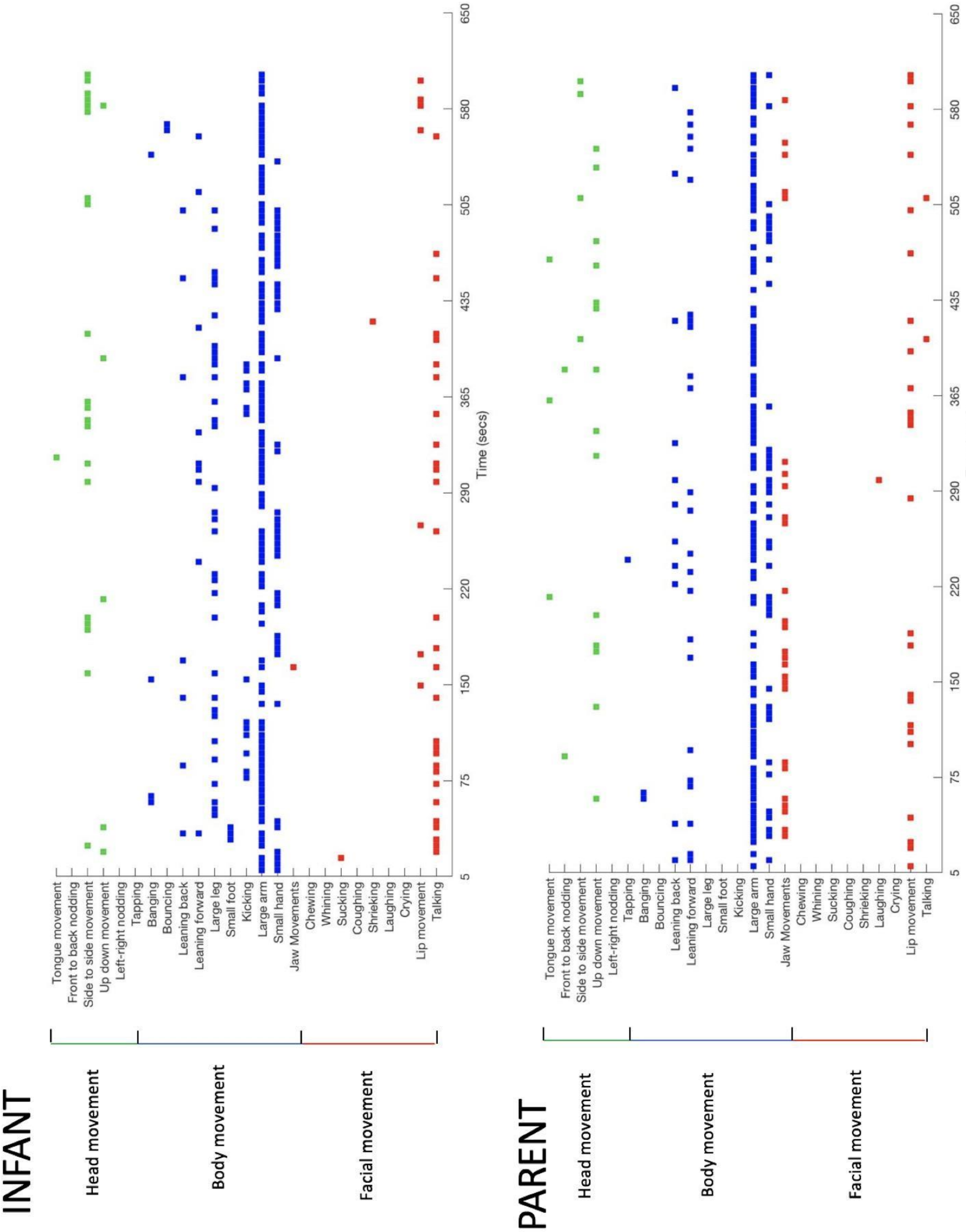

Figure S2. Example of the coding procedure by time and motion type for one infant-parent pair.

2.2 Results

As shown in Figure S3a, at least one type of motion was present in over 95% of the time epochs during all tasks. A more detailed breakdown of the occurrence of each class of motions is provided in Tables S3-S5. We also noted that the motions overlapped substantially with each other in time, and a single type of motion occurred relatively infrequently. There was an average of 34.88% (SD = 9.16%) and 76.04% (9.45%) overlap in time between different motions for adults, and an average of 71.77% (SD=13.96%) and 80.64% (5.09%) for infant, for SP and JP respectively (Figure S3b).

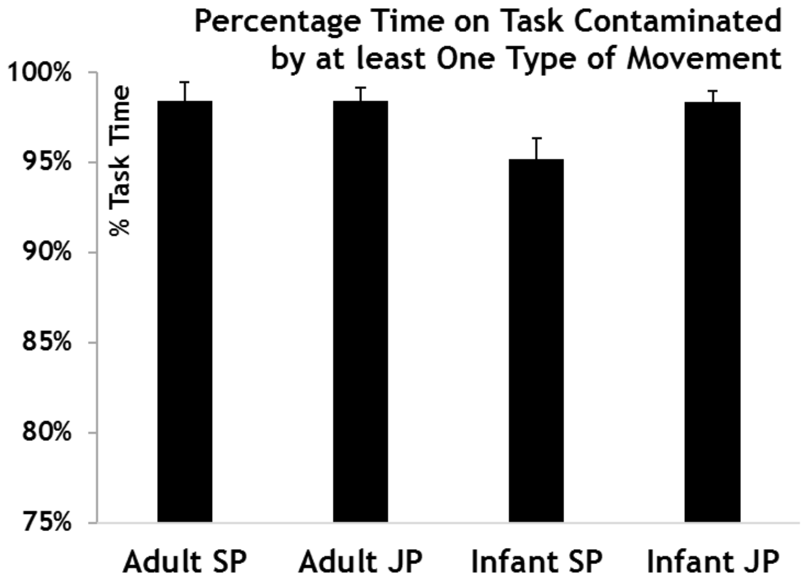

Figure S3a. Proportion of the time when at least one movement was observed. Error bars show the standard error of the mean. SP – separate play, JP – joint play conditions.

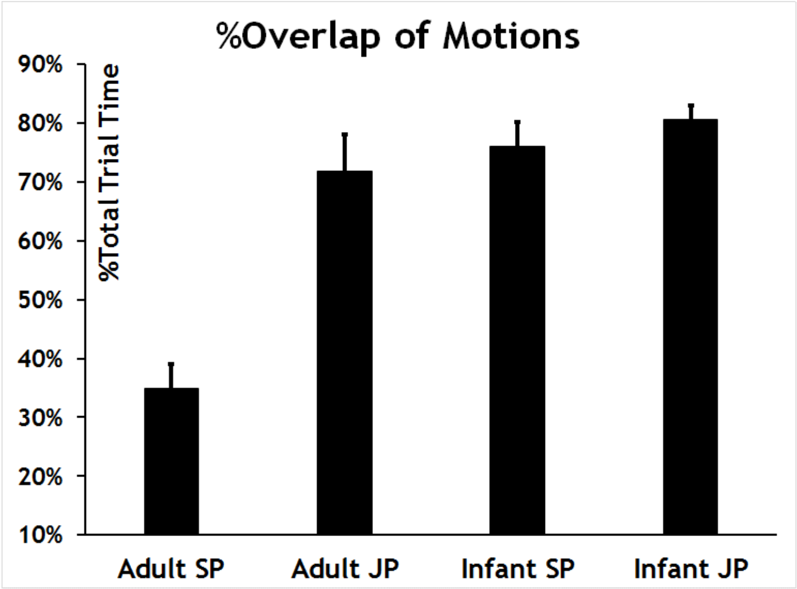

Figure S3b. Percentage overlap in time of two or more motions for the infants and adults in the two play conditions.

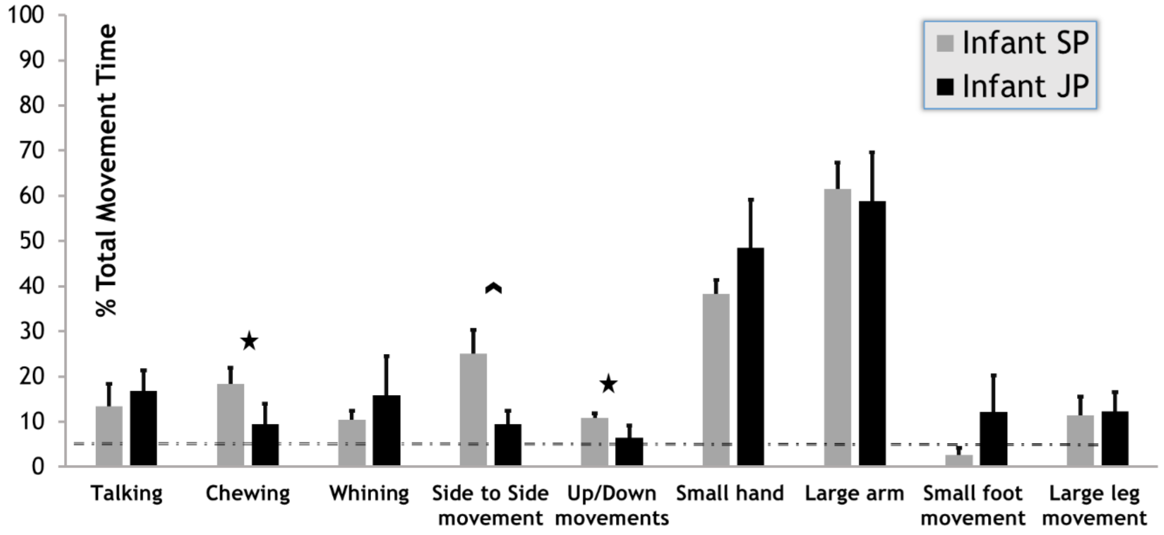

*Figure S3c. Prevalence of infant motions occurring over 10% of the time in each experimental condition. *p<.05; ^p=.051 (uncorrected).*

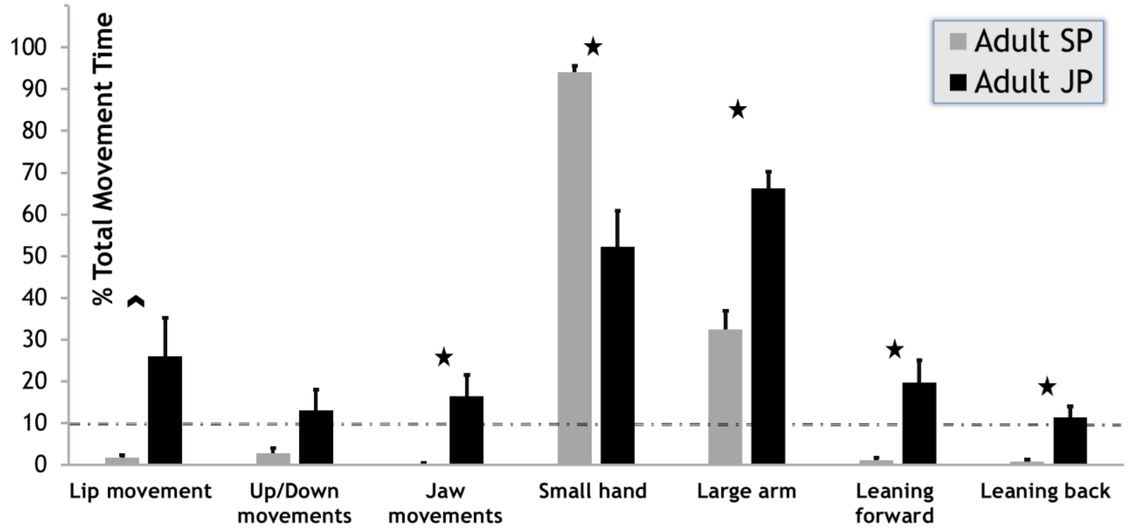

*Figure S3d. Prevalence of adult motions occurring over 10% of the time in each experimental condition. *p<.05, ^p=.054 (uncorrected)*

To assess whether the total prevalence of motion differed between participants or conditions, a 2 (Participant: Adult, Infant) x 2 (Play Condition: Separate, Joint) ANOVA was conducted. The results showed no significant main effect for Participant, $F(1,16) = 3.31$, $p=.09$, or for Play Condition, $F(1,16) = 2.92$, $p=.11$. The interaction between Participant and Play Condition was also non-significant, $F(1,16) = 3.01$, $p=.10$. Therefore, we observed equal proportions of motion contamination across both play conditions, and for infants' and adults' artifacts, when all motion types were pooled together.

Comparative statistics were computed to identify the most commonly occurring motions in each condition; and to assess whether the prevalence of these most common motions differed between social (JP) and non-social (SP) conditions. Commonly occurring motions were defined

as motions that occurred at least 10% or more of the time (regardless of whether they occurred individually, or in conjunction with other motions).

For infants, the most common movements included Talking, Chewing, Whining, Side-to-Side Neck movements, Up and Down Neck movements, Small Hand, Small Foot, Large Arm and Large Leg movements (Figure S3c). The frequency of each one of these movements was compared between the two play conditions using paired t-tests. Only Chewing, and Up and Down Neck Movements differed significantly in prevalence between Separate and Joint Play conditions, $t(4) = 3.68$, $p<.05$ and $t(4) = 2.81$, $p<.05$, respectively (p-values uncorrected, no significant difference for either after Bonferroni correction). There was also a marginal difference between conditions for Side to Side Movements ($t(4) = 2.76$, $p=.051$). More of these infant motions occurred during Separate than during Joint Play. The remaining motions showed no statistical difference in their frequency of occurrence between the two play conditions: talking, whining, small hand movements, large arm movements, small foot movements, large leg movements (all $p>.25$).

For adult participants, the most common motions were: Up-Down Neck, Lip, Jaw, Small Hand, Large Arm, Leaning Forward and Leaning Back movements (Figure S3d). From inspection of Figure S3d, the prevalence of all these motions indeed differed between social and non-social conditions, apart from Up-Down Neck Movements. In general, motion was more frequently observed during JP than SP, with the exception of small hand movements that were more frequent during SP. This pattern was confirmed by a series of paired t-tests (p-values uncorrected); Lip Movement: $t(4) = 2.70$, $p =.054$; Up and Down Neck Movements: $t(4) = 1.92$, $p=.13$; Jaw Movements: $t(4) = 3.10$, $p<.05$; Small Hand Movements: $t(4) = 4.19$, $p<.05$; Large Arm Movement: $t(4) = 5.59$, $p<.01$; Leaning Forward: $t(4) = 3.40$, $p<.05$; Leaning Back: $t(4) = 4.17$, $p<.05$. However, after applying a (conservative) Bonferroni-correction of p-values (alpha = .05), only Large Arm Movements showed a significant increase in prevalence for the Joint Play condition.

2.3 Descriptive Statistics for Motion Prevalence

Tables S3-S5 summarise the relative frequency of occurrence of facial, body and head movements observed in both groups of participants and across both conditions. In each table, the percentage occurrence for each motion type out of total time that participants spent on the task is reported.

Table S3. Descriptive statistics for facial motions. The table reports the mean percentage of the total time (brackets standard error, SE) during which these motions were observed.

	**Facial Motion Statistics (%)**									
	Talking	Lip mvmt	Chewing	Sucking	Coughing	Shrieking	Laughing	Crying	Whining	Jaw mvmt
Adult SP	0.00 (0.00)	1.66 (0.59)	0.00 (0.00)	0.00 (0.00)	0.22 (0.22)	0.00 (0.00)	0.32 (0.20)	0.00 (0.00)	0.00 (0.00)	0.22 (0.22)
Adult JP	4.45 (4.07)	25.95 (9.31)	0.31 (0.31)	0.00 (0.00)	0.00 (0.00)	0.00 (0.00)	1.48 (0.71)	0.00 (0.00)	0.00 (0.00)	16.38 (5.10)

	Facial Motion Statistics (%)									
	Talking	Lip mvmt	Chewing	Sucking	Coughing	Shrieking	Laughing	Crying	Whining	Jaw mvmt
Infant SP	13.38 (4.95)	1.40 (1.05)	18.32 (3.68)	3.25 (3.10)	0.49 (0.34)	1.59 (0.55)	0.16 (0.16)	1.09 (1.09)	10.49 (1.90)	1.43 (0.63)
Infant JP	16.86 (4.53)	2.82 (1.41)	9.40 (4.61)	6.81 (4.00)	0.54 (0.54)	1.30 (0.71)	4.11 (1.86)	0.00 (0.00)	15.82 (8.60)	0.16 (0.16)

Results of the t-test analyses suggest that, although there was no significant difference in infant talking between the JP and SP conditions, there was a significant difference in adult lip movement between the two conditions, which might be explained by adults using the lip movement in the JP condition as a way of communicating silently with the infant. Significant differences between infant chewing was observed between conditions, which might be explained by the infants using chewing as a form of comfort when they could not visibly see the adult behind the screen during SP.

Table S4. Descriptive statistics for body motions. The table reports the mean percentage of the total time (brackets standard error, SE) during which these motions were observed.

	Body Movement Statistics (%)										
	Small hand	Large arm	Foot mvmt	Kicking	Small leg	Large leg	Bouncing	Banging	Tapping	Leaning forward	Leaning back
Adult SP	94.06 (1.52)	32.42 (4.46)	0.00 (0.00)	0.00 (0.00)	0.00 (0.00)	0.00 (0.00)	0.00 (0.00)	0.28 (0.18)	2.28 (2.14)	1.03 (0.64)	0.76 (0.41)
Adult JP	52.27 (8.53)	66.19 (4.11)	0.00 (0.00)	0.00 (0.00)	0.00 (0.00)	0.00 (0.00)	0.00 (0.00)	0.84 (0.38)	3.80 (1.09)	19.62 (5.40)	11.41 (2.49)
Infant SP	38.19 (3.21)	61.50 (5.90)	2.58 (1.64)	2.47 (2.28)	0.65 (0.40)	11.47 (4.01)	2.98 (2.14)	6.17 (1.02)	0.00 (0.00)	8.09 (2.10)	4.10 (1.57)
Infant JP	48.41 (10.65)	58.79 (10.90)	12.09 (8.20)	2.50 (1.95)	6.52 (4.55)	12.34 (4.19)	0.88 (0.41)	2.24 (1.21)	0.19 (0.19)	5.24 (2.04)	3.50 (1.03)

The most prominent body movements included: adult small hand movement during JP condition (52.27%); adult small hand movement during SP condition (94.06%); infant small hand movement during SP condition (38.19%); infant small hand movement during JP condition (48.41%); infant small foot movement during the JP condition (12.09%), adult large arm during SP condition (32.42%), adult large arm during JP condition (66.19%), infant large arm during JP condition (58.79%), infant large arm during SP condition (61.50%), and adult learning forward during JP condition (19.62%).

As both conditions involved play with intricate toys that could be waved around, it was no surprise to see that for both the adult and infant, small hand and large arm movements were the most prevalent. There were, however, significant differences observed between the adult small hand movement in both conditions, which might be explained by the adults spending more time watching the infant play during the JP task, rather than playing with the toy themselves. It was further not surprising to see a difference in the adult large arm movement, which might be explained as in the JP condition the adult participants were frequently observed reaching forward

to comfort the child or present them with a toy. The adult participants were less likely to create large arm movements in the SP task and instead were observed producing small hand movements for almost the entire time, with the adults' small hand movements demonstrated as the most prevalent motion across all conditions.

Table S5. Descriptive statistics for head motions. The table reports the mean percentage of the total time (brackets standard error, SE) during which these motions were observed.

	**Head Movement Statistics (%)**					
	L-R nodding	Tongue mvmt	F-B nodding	Side-Side mvmt	Up-Down mvmt	Arching back
Adult SP	0.00 (0.00)	0.22 (0.22)	0.33 (0.33)	3.10 (1.67)	2.69 (1.35)	0.00 (0.00)
Adult JP	0.00 (0.00)	2.63 (0.91)	0.91 (0.37)	1.63 (0.74)	12.98 (5.03)	0.00 (0.00)
Infant SP	1.15 (0.56)	0.17 (0.17)	0.00 (0.00)	25.01 (5.35)	10.79 (1.12)	1.53 (1.15)
Infant JP	0.27 (0.27)	0.34 (0.21)	0.00 (0.00)	9.40 (3.07)	6.49 (2.61)	0.20 (0.20)

The most prevalent head movements observed were the adult up-down movement during the JP condition (12.98%); the infant side-side movement during the SP condition (25.01%); the infant up-down movement during the SP condition (10.79%). In the JP condition, the paired t-test analyses suggest that the adult up-down head movements were observed significantly more than during SP, which may be due to the JP task enabling adults to communicate and interact with the infants through actions as the adults had been briefed to remain silent. Frequently, the adult was seen mimicking actions the infant produced to entertain them. A significantly greater percentage of time was observed in the infant side-side head movement and up-down movement in the SP condition over the JP condition, and this may be due to the child searching for the adult participant or other adult figures in the room.

2.4 Discussion

In this behavioural pilot study, we assessed the prevalence of motion in adult-infant dyads during social and non-social naturalistic play paradigms. We observed that motion occurred >95% of the time, for both the infants and adults, and in both types of play settings. Interestingly, whilst the adults' gestural motions (e.g. arm movements) tended to increase in frequency for social as compared to non-social play, the infants showed less looking around and chewing. This may be attributable to the fact that infants were more engaged and less distracted during the social play with their mothers. For such datasets in an EEG experimental context, it would not be feasible to adopt a simple approach of rejecting (excluding) all motion-contaminated data, as this would entail losing an unacceptably high proportion of the data. However, before artifact removal methods (such as ICA or CCA) can be effectively applied to the EEG signal, it is first necessary to understand the exact distortion that these motions produce. Accordingly, the main study attempted to document the topographical and spectral properties of each of the most prevalent (frequently occurring) types of motion that were observed in the pilot.

3 Main experiment

3.1 Individual infants' scalp topographies during Resting State and Motion

Figure S4. Scalp topographies of infant EEG power [uV^2/Hz] for RS and all movement types:

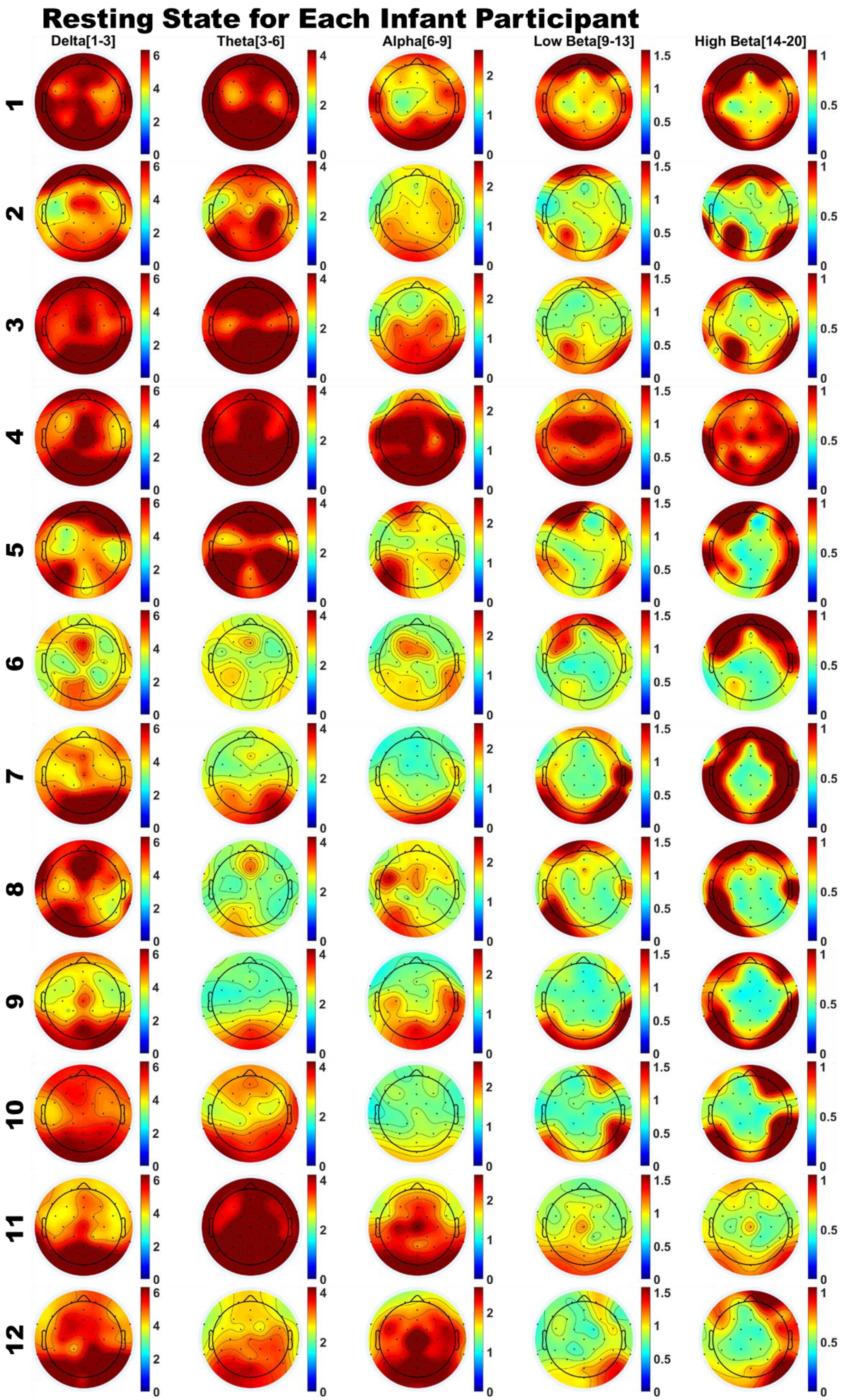

(a) Resting State

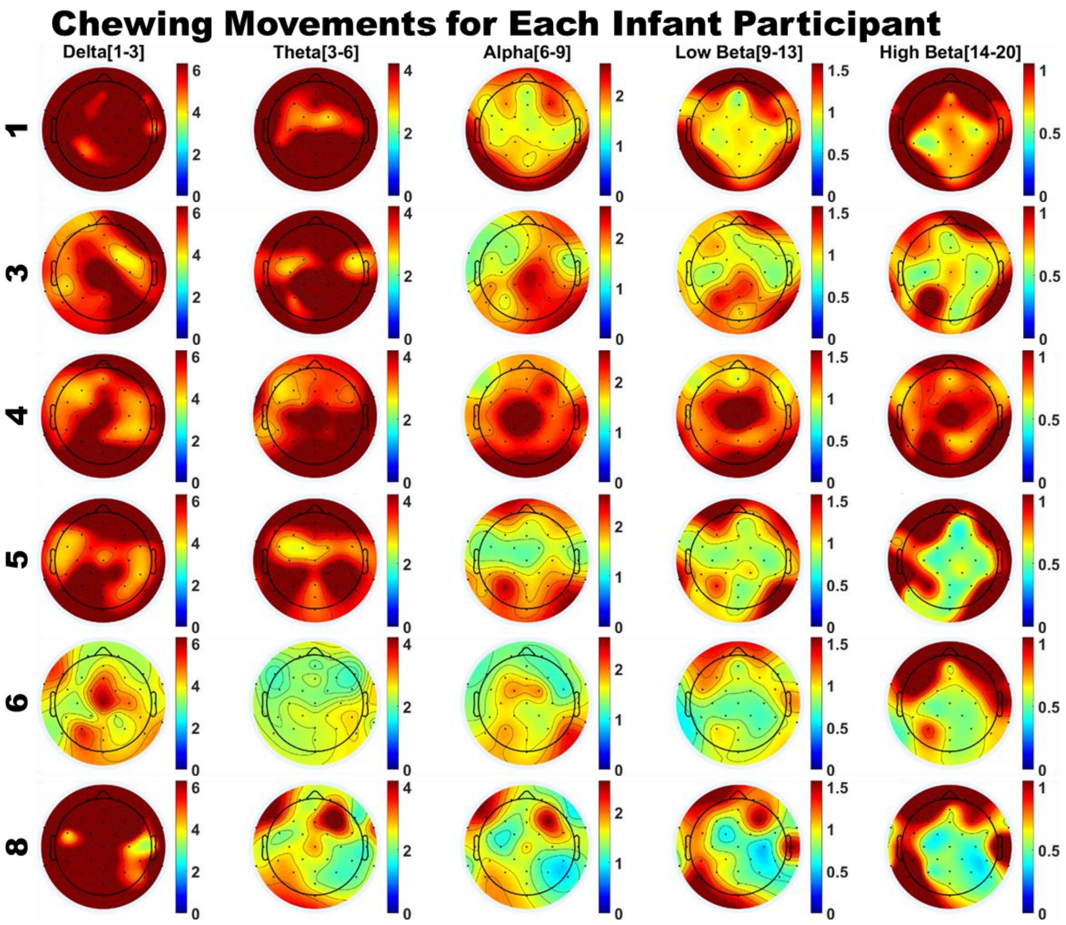

(b) Chewing Movements

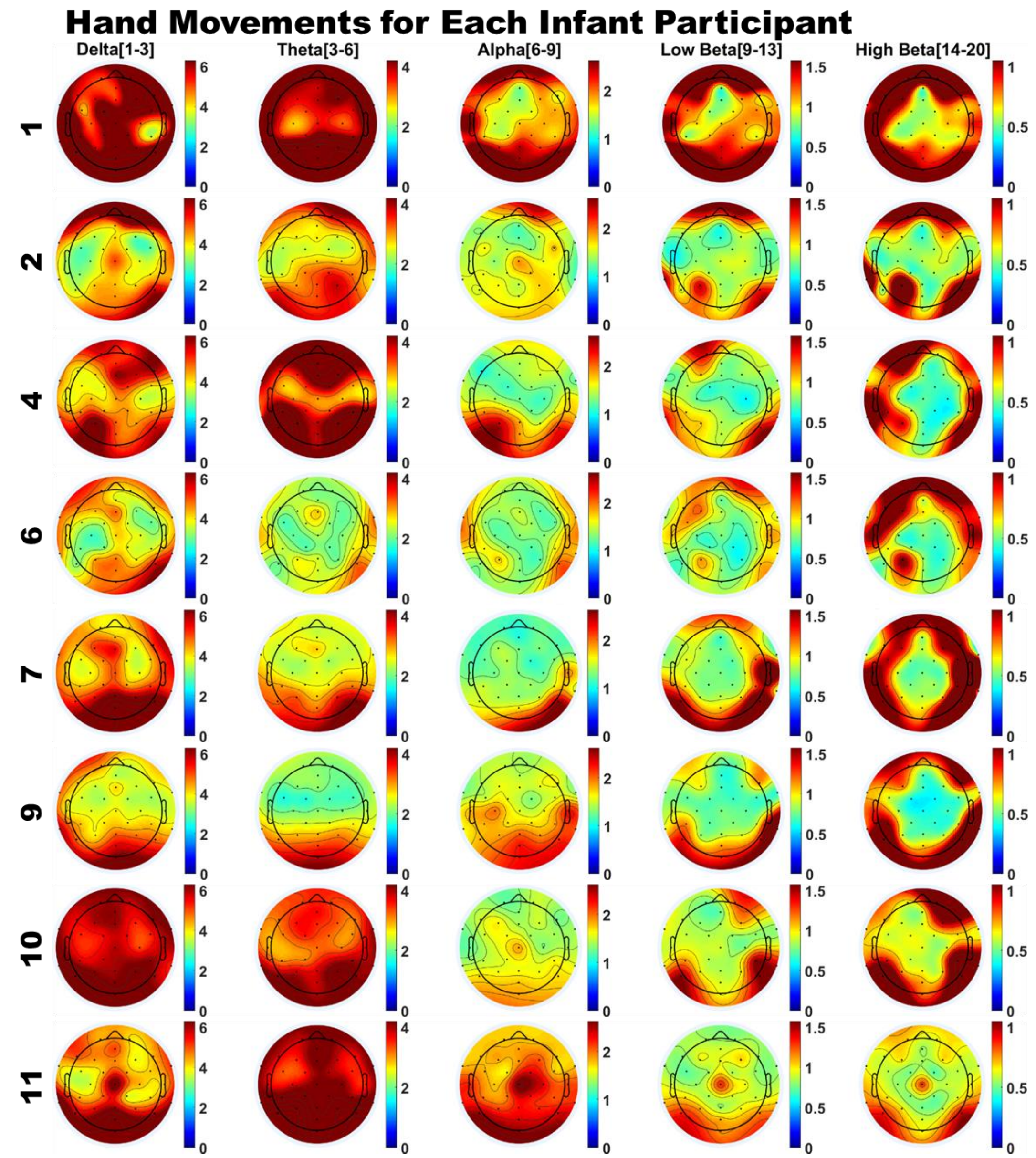

(c) Limb Movements: Hand

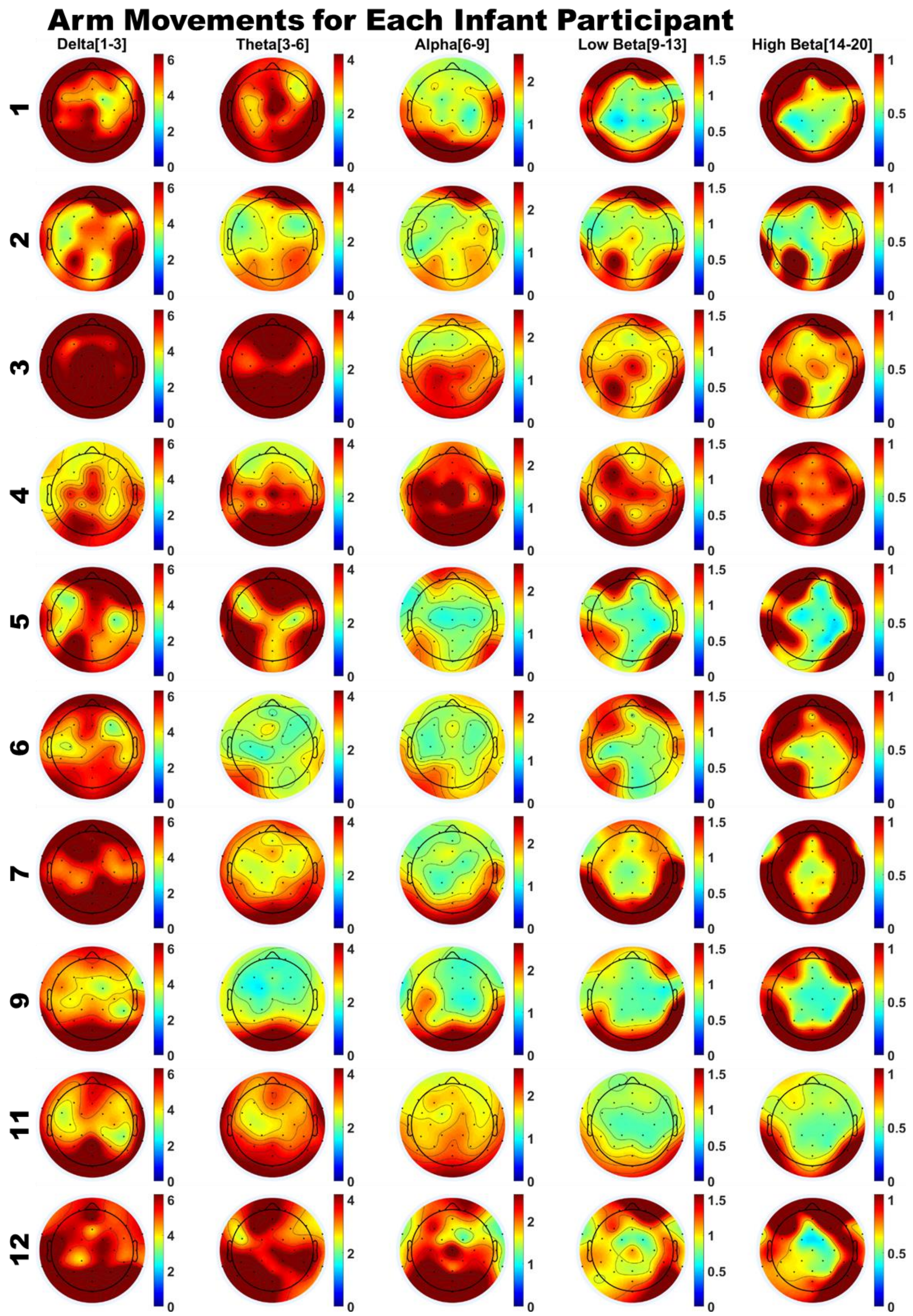

(d) Limb Movements: Arm

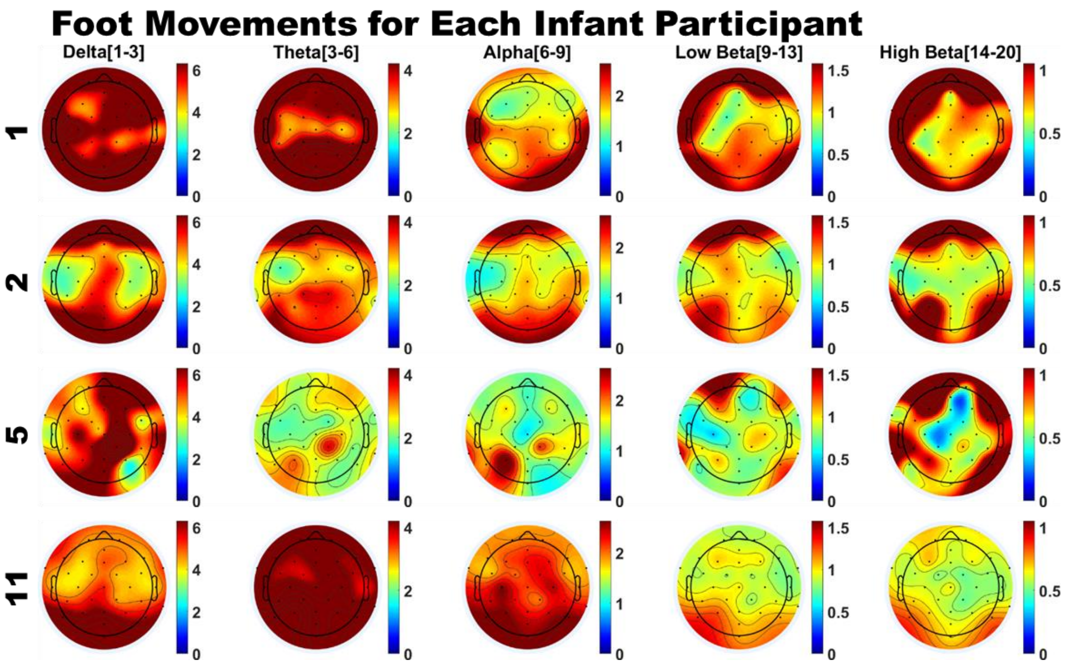

(e) Limb Movements: Foot

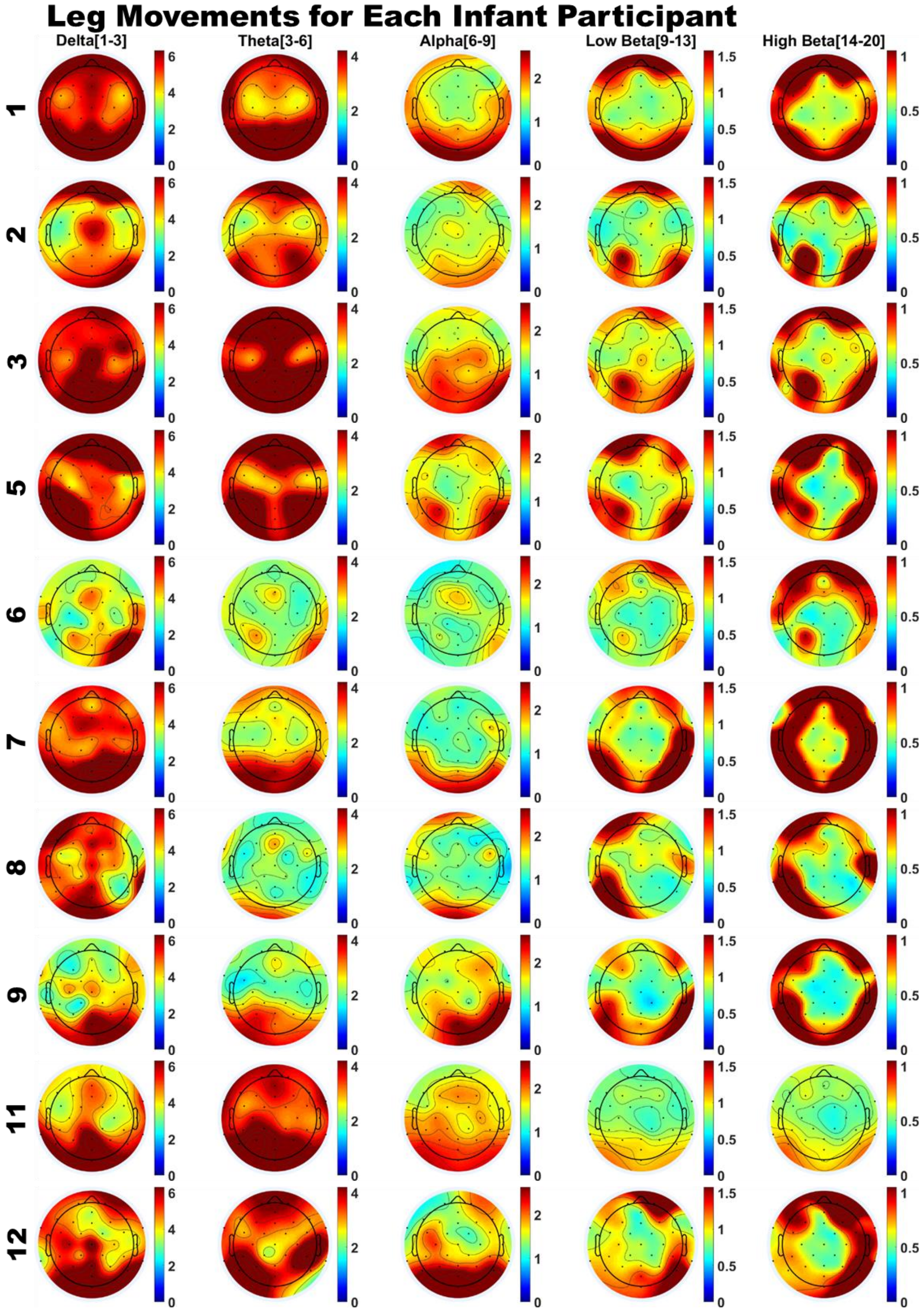

(f) Limb Movements: Leg

3.2 Statistical Stratification to assess for effects of data duration differences across conditions

To assess whether differences in the data duration between the motion and resting state conditions (see Table II in main text) introduced any systematic biases, we conducted an additional statistical stratification analysis for the Jaw and Arm conditions, which were the only two motions that differed significantly from resting state.

For each participant, a continuous segment of the same duration as the shorter dataset (motion or resting state) was selected with a random onset from the longer dataset (note that in some participants, the motion recording was longer than the resting state, i.e. Jaw motions for infant 5). Next, EEG power analysis was conducted using the same procedure as described in the main text, Section 2.4. These two steps were repeated 1000 times. The power spectra for each channel were then averaged across the 1000 iterations. Finally, a cluster-based permutation analysis was conducted on the averaged power spectra following the same protocol as described in Section 2.4 in the main text.

The results from the cluster-based permutation analysis of stratified data from Arm-related motion effects are virtually identical to the main analysis (Figures S5a and S5b*)*. Due to the data reduction, the sensitivity of the cluster permutation test decreases, and we noted higher variance in the average power spectra in each condition, and lower test statistics for the significant cluster. For Jaw-related motion, the stratified data yielded no significant effects, although the spectral patterns were similar to the main analysis (Figure S5c and S5d). This non-significance was most likely due to a lack of sufficient power in the reduced subsample.

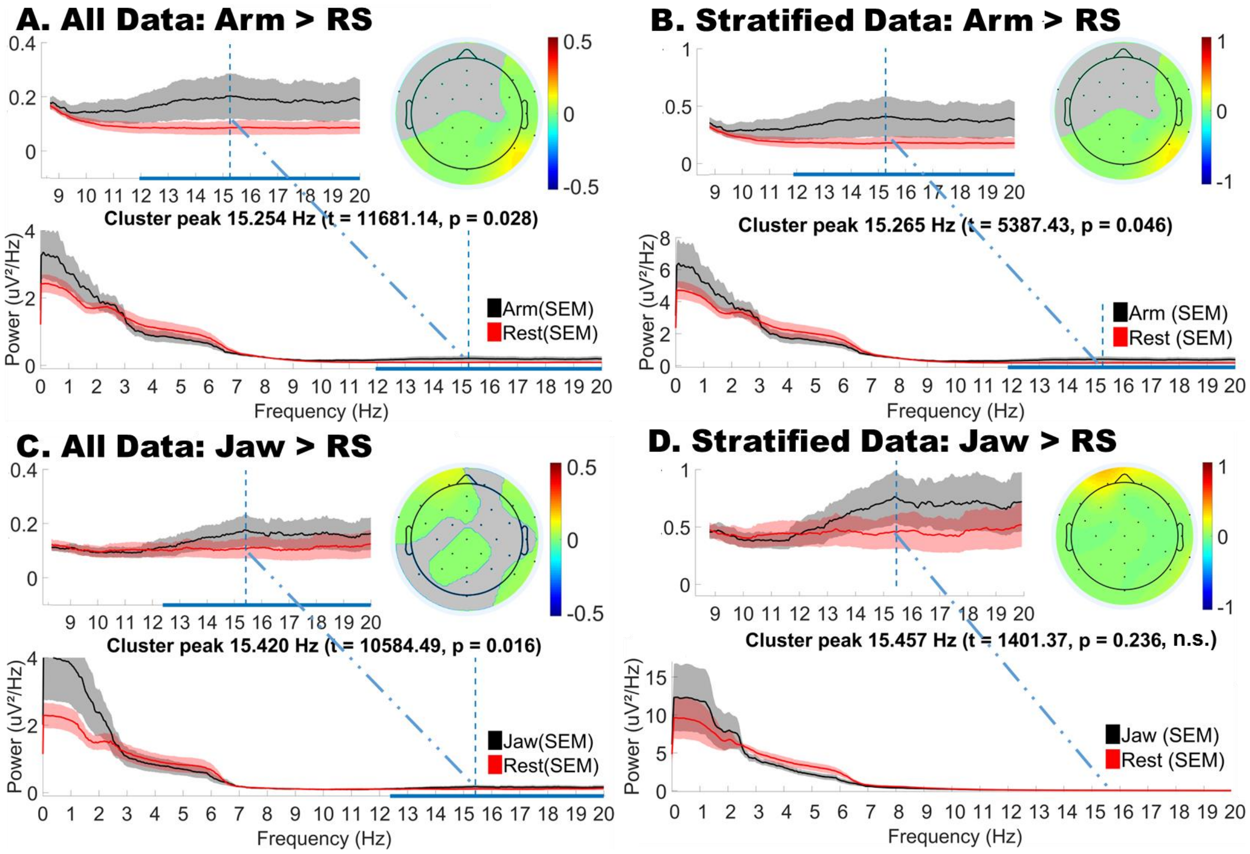

Figure S5. Topographical and spectral differences in infant EEG power for Arm Movements (A and B) and Jaw (C and D) relative to resting state. A and C: Original comparison with including all data. B and D Same comparison after stratification. The line plots show the power spectra for motion (black line) and resting state (red line), with their respective standard error of the mean (SEM) in coloured shading. The horizontal blue line on the x-axis indicates the frequency range of significant differences in power were observed. The vertical blue line shows the peak difference in power and the headplots above show the scalp topography of the cluster at the peak difference in power. The grey areas in the headplots (a,b,c) show non-significant difference. The color bar demonstrates differenced power.

3.3 ICA Analysis

We used Arm motion as a case study of the effectiveness of ICA in detecting motion-related components for infant EEG cleaning. Ten infants contributed EEG data for this analysis.

A general word of caution in the application of ICA without a devoted channel (i.e. EOG) – researchers normally decide a-priori whether they are selecting a specific component of interest (i.e. ICs for frontal alpha only) and removing all other components (which may contain both artifactual but also neural activity); or whether they will take a more conservative approach and remove components that are certain to represent artifactual activity only. The latter approach is likely to result in lower distortion of the reconstructed neural EEG signal although it is also more likely to retain artifactual activity. The following approach was adopted for this analysis:

Step (1) ICA (Matlab function *runica()*, ran from EEGLAB) was performed on the raw EEG signal to remove ICs which clearly represented eye-movements (blinks and square-wave shaped saccades). In five of the ten datasets, blink and saccade ICs were co-identified by two researchers experienced in adult EEG (*not* independently). Blinks were mostly stereotypical in this cohort. An example is given in Figure 4 in the Results section below. Note that in the main analysis reported in this manuscript, ICA was not used in the pre-processing pipeline. Rather, blinks and saccades were removed manually by eye. After ICA removal, the continuous EEG signal was segmented into epochs containing only either Resting State (RS) or Arm movements respectively.

Step (2) The topographical and spectral differences between Arm and RS data were compared using the exact steps detailed in the main manuscript. At this stage, we expected to see a similar pattern of differences to those reported in the main manuscript.

Step (3) Next, a second ICA cleaning step was performed only for the Arm movement segments. Using the spectral differences maps produced in Step (2) as a guide, additional ICs specifically relating to Arm movement were removed from infants' EEG data. The final cleaned Arm movement data were again compared with rest using cluster-based permutation analysis to identify any remaining topographical and spectral differences. If the second ICA step was successful in removing Arm movement artifacts, there should be no remaining differences.

Note that ICA was not conducted *separately* for Arm movement and RS segments for three reasons: First, the ICA would be less successful for shorter amounts of data. Second, it would have been very difficult to identify arm movement ICs in a continuous recording as there would be no clear onset and offsets to signify the motion and guide component detection. And finally, as we ran ICA for eye-movement detection on the continuous EEG recording and removed the blink and saccade components, we had already reduced the number of available dimensions in the data. Given that by default ICA returns as many independent components (ICs) as there are electrodes, forcing another ICA would produce a new full set of ICs which might cause some of the original ICs from the first run to be spilt over two or more new ICs. This could cause one IC containing artifactual activity to be spread over more ICs after the second run. The more artifactual ICs there are, the more likely it is that they would also contain non-artifactual activity, which makes the separation between the two even more challenging.

Results after Step 2 (basic ICA removal of eye movements artifacts):

Figure S6a (left panel) shows the original results in the main text for the spectral differences between the Arm movements and Resting State data, with the cluster-permutation significance test. Figure S6b (right panel) shows the same comparison with eye-movement components removed via ICA.

As expected, the original (left) and IC-corrected (right) clusters yielded highly similar results, with significant differences observed in the beta bands in nearly identical topographical regions. This analysis confirmed that – in the case of eye movement artifacts - ICA performed similarly to our original method of manual eye-movement artifact removal.

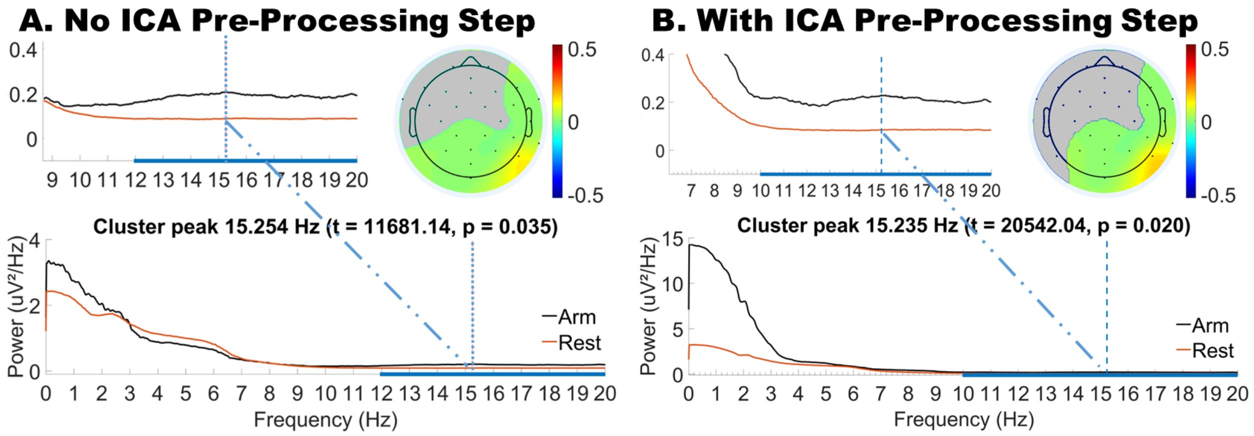

Figure S6. Topographical and spectral differences in infant EEG power for Arm Movements relative to resting state. The line plots below show the power spectra for motion (black line) and resting state (red line). The horizontal blue line on the x-axis indicates the frequency range over which significant differences in power were observed. The vertical blue line shows the frequency of peak difference in power, and the headplots above this show the scalp topography of the cluster at the peak difference in power. The grey areas in the headplots show non-significant difference. The colour bar indicates differenced power. A. Original comparison with blinks removed manually from continuous EEG recording. B. Same comparison with blinks removed as ICA component.

<u>Results after Step 3 (further ICA removal of Arm movement artifacts):</u>

Figure S7 shows an example of the Arm movement components (IC6, IC11, IC14 and IC25) identified for one infant. Despite being guided by the topographic difference maps (Figure S6), it was extremely challenging to identify ICs that contained only an Arm movement component, both spectrally (i.e. where the peak frequency would be) but also topographically (we assumed it would be most prominent in peripheral temporal and tempo-parietal channels), due to the lack of previous research. Another concern was that the ICA was unable to identify a single Arm component - Arm movements were spread over many components. So although all care was taken to not remove any components that looked similar to classical resting state (i.e. if they contained a central alpha peak), by removing some of the ICs that we thought contained arm movements, we likely removed other aspects that were not related to Arm movements, and thus overcorrected compared to RS.

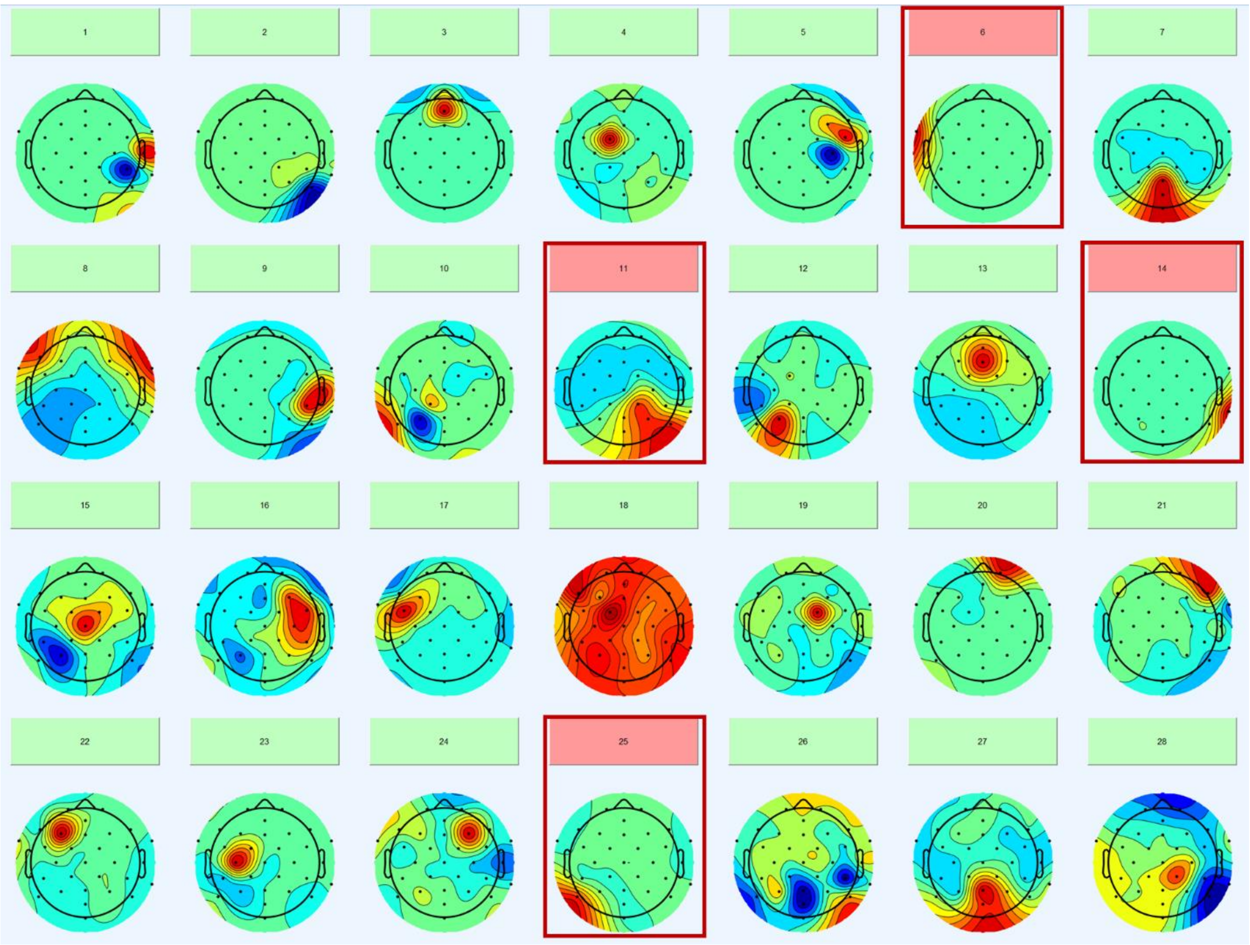

Figure S7. A screenshot of the IC rejection stage in EEGLAB (Matlab) for an example infant participant, available after running ICA in EEGLAB. Each one of the spectral head heatmaps (nose is above; channels are represented by dots on the head) correspond to a single IC. The red and blue colours show positive and negative peaks for that component. The user is able to click on each IC (examples are given in Figure S8a,c and f) and inspect the power density spectrum for that component, as well as the power distribution of the time course for that IC for each time window of the Fast Fourier transform (FFT time window here was 1.5 s – the default selection of the runica() function). The ICs marked in a red rectangle were selected for rejection for this infant.

Figure S8 (the right panel) shows that the cluster of spectral differences between the Arm movement and RS segments was not significant after the secondary removal of Arm movement-related ICs. It may be observed that the second ICA step was effective in removing the vast majority of artifactual activity from the Arm movement data. However, the Arm movements were spread over a number of IC, which may have led to either incomplete removal of Arm movement-related activity, or removing some activity not related to Arm movements which was classified within the same components. Here, we were fairly confident that all components removed contained Arm movements (for example, they were consistent with similar components related to arm movements in adults – see Figure S9e). However, we cannot be certain that they contained Arm movement *only*.

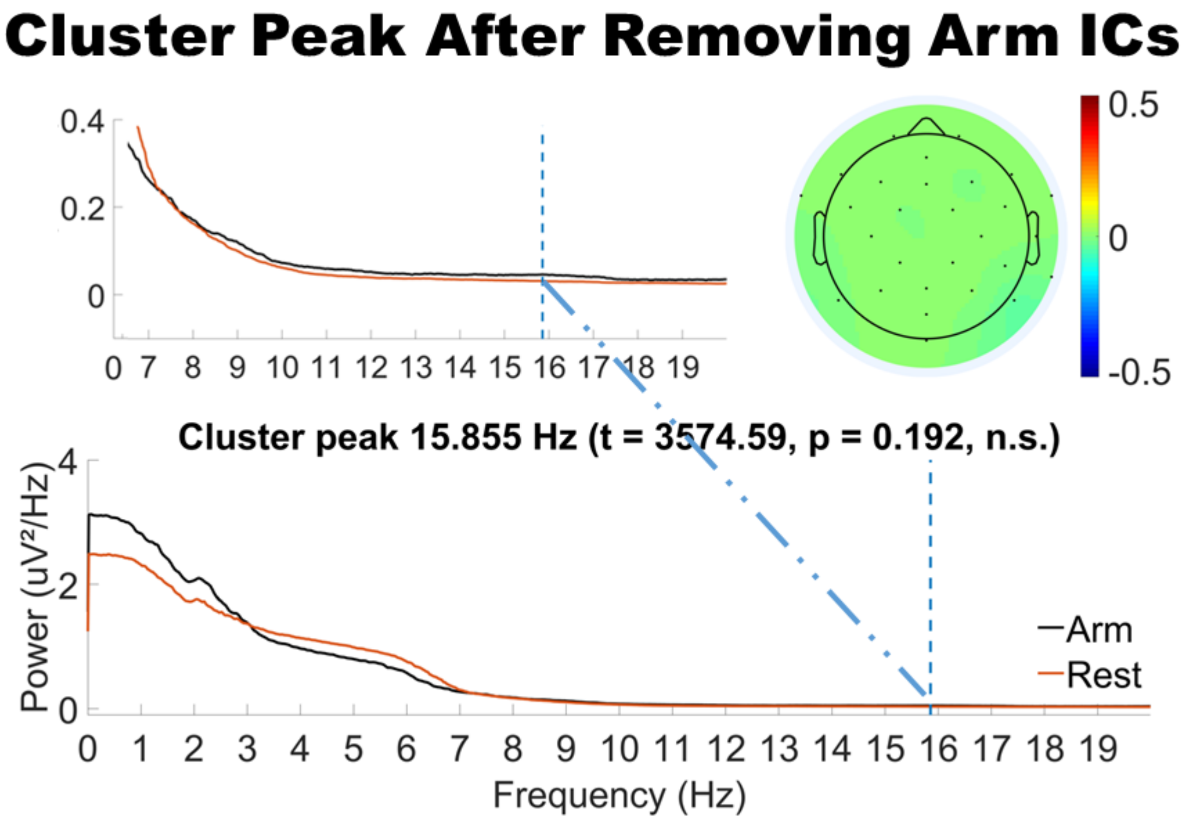

Figure S8. Topographical and spectral differences for the Arm Movements relative to resting state after the arm-movement related ICs had been removed. The line plots below show the power spectra for motion (black line) and resting state (red line). The horizontal blue line on the x-axis indicates the frequency range over which significant differences in power were observed. The vertical blue line shows the peak difference in frequency and the headplot above this shows the scalp topography of the cluster at the peak difference in frequency. The colour bar indicates differenced power.

Figure S9 demonstrates the power spectra and time course of some of the more common components identified in this analysis. Saccades and blinks were identified in all infants but one, and had a very stereotypical pattern across infants in both spectral and time domains. Interestingly, in 7/10 infants, Arm movements were identified in 2-3 ICs. In the remaining three infants, decomposition was less successful, with Arm movement-like activity observed in four, or even five components (as in Figure S7). Finally, we made an intriguing observation that some of the infant ICs were very similar to ICs obtained from an adult EEG recording of a completely unrelated task in which the adults used an aviator joystick in their right hand to respond in a simple task (here we are showing the author's own unpublished data on a perceptual decision-making task designed by Rogers & Davis, 2009). The components found in the adults related to the joystick movements were very similar in their spectral and temporal profile to some independent components observed in our infant dataset (Figure S9e).

Figure S9. Examples of independent components for blinks, saccades and arm movement in a representative infant. *Plots (a) – (d)* are from the same infant; *plot (e)* includes another infant and two adult participants from an unrelated experiment (author's own unpublished data).

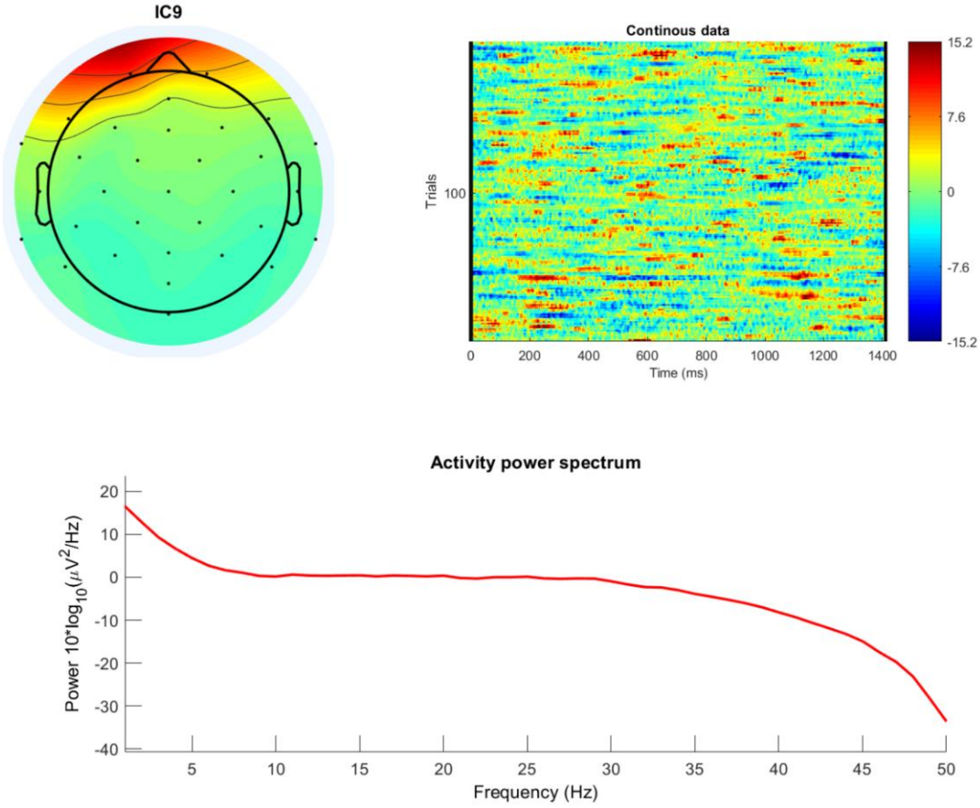

a. *Blink-related IC.*

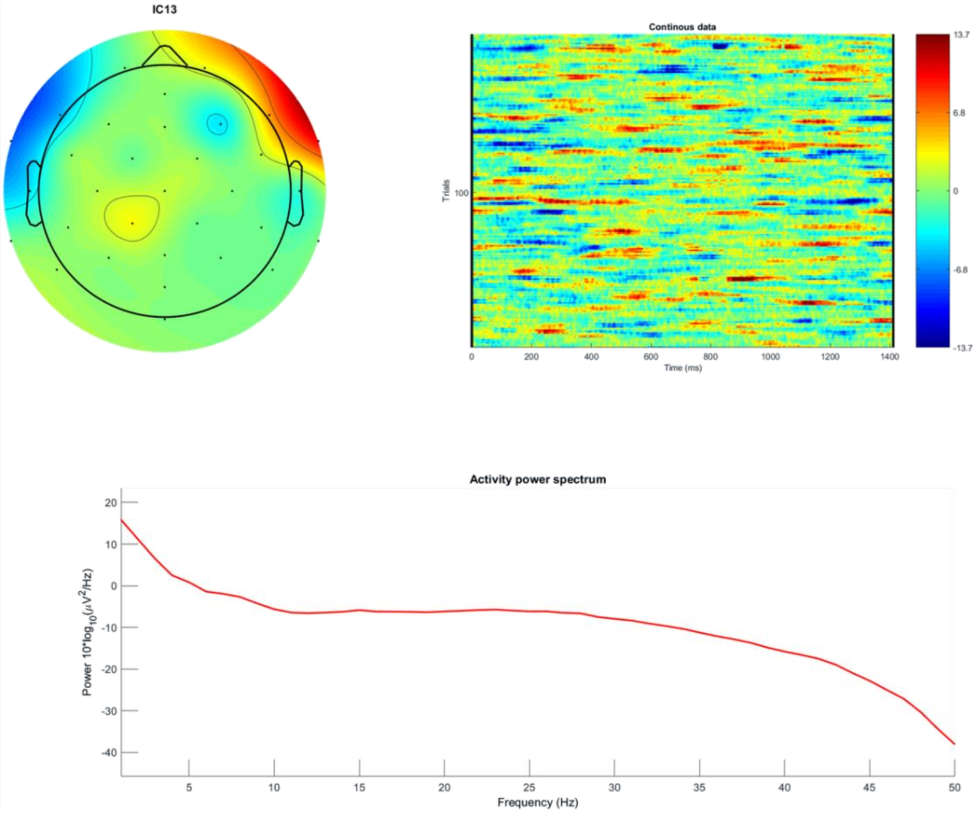

b. *Saccade-related IC.*

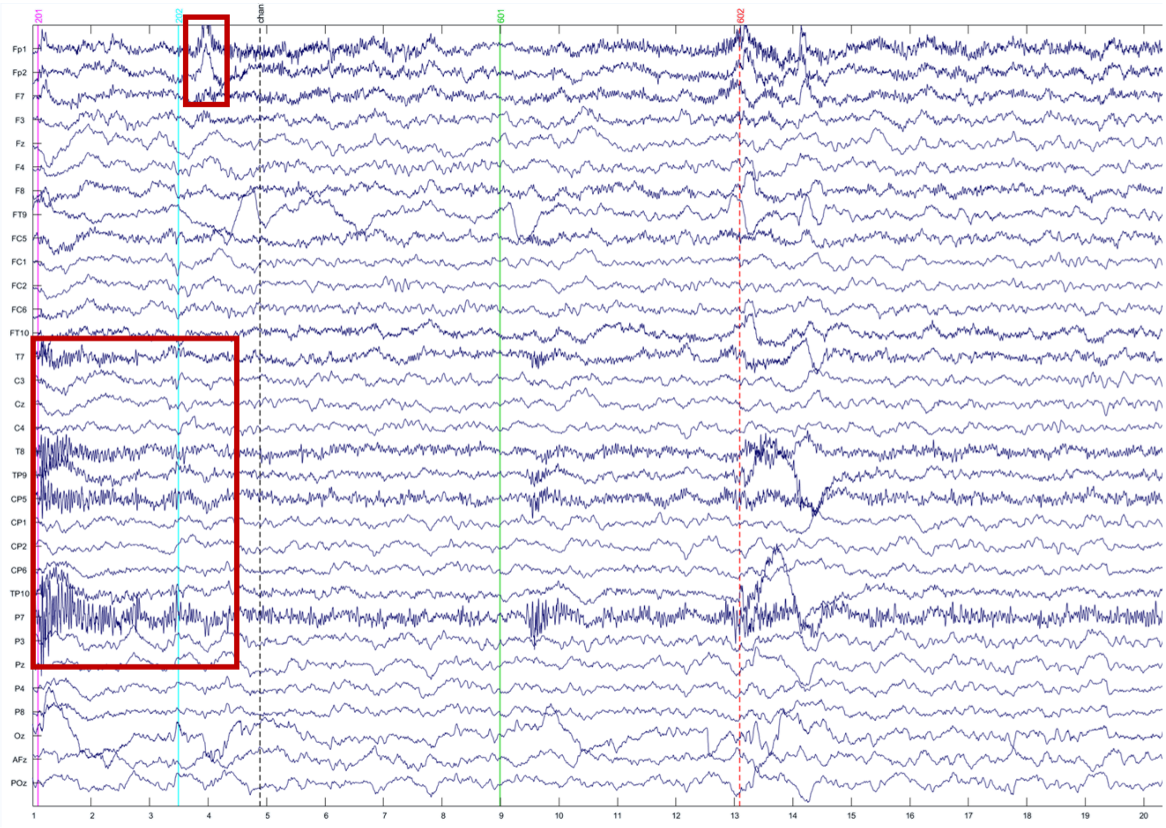

c. *Raw EEG trace by channel. The eye-blinks are highlighted in red in the top two channels and Arm movements are highlighted in red in many of the middle channels.*

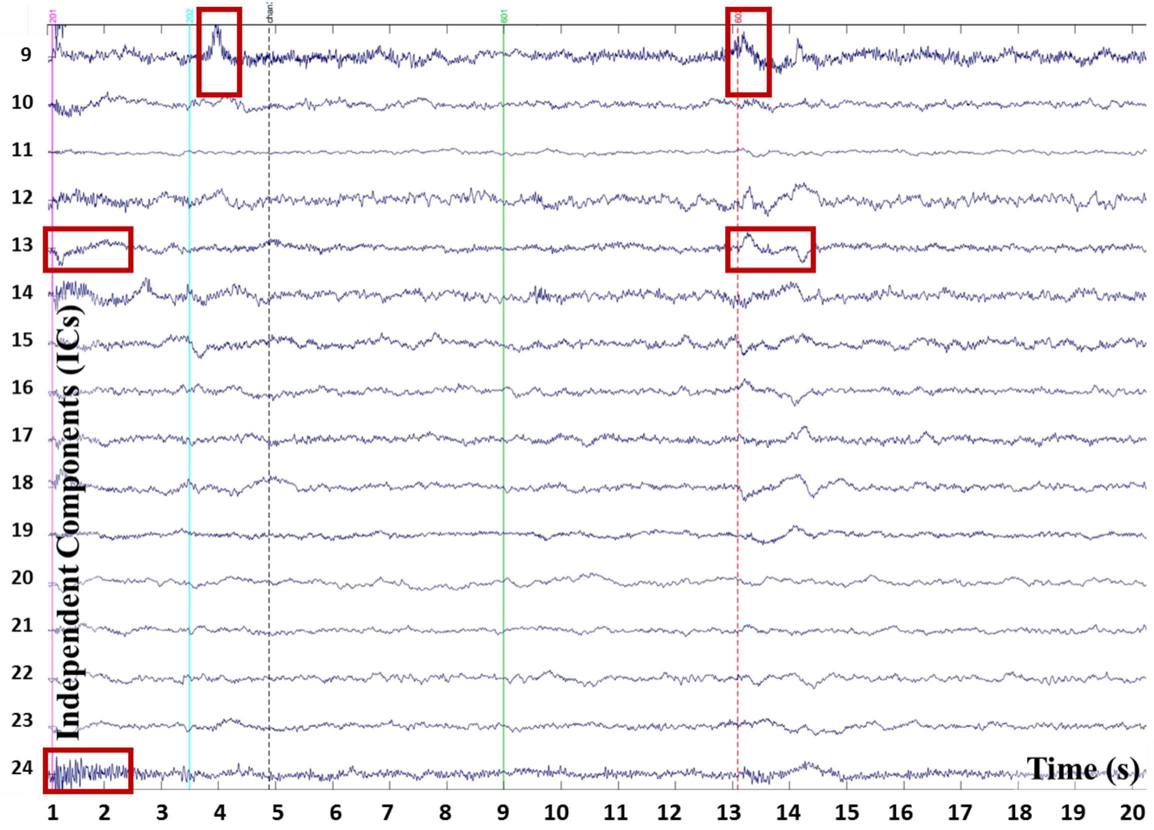

d. *The time-series for IC9 to IC24. Blinks (IC9), Saccades (IC13) and Arm movements (IC24) components are highlighted in the red rectangles.*

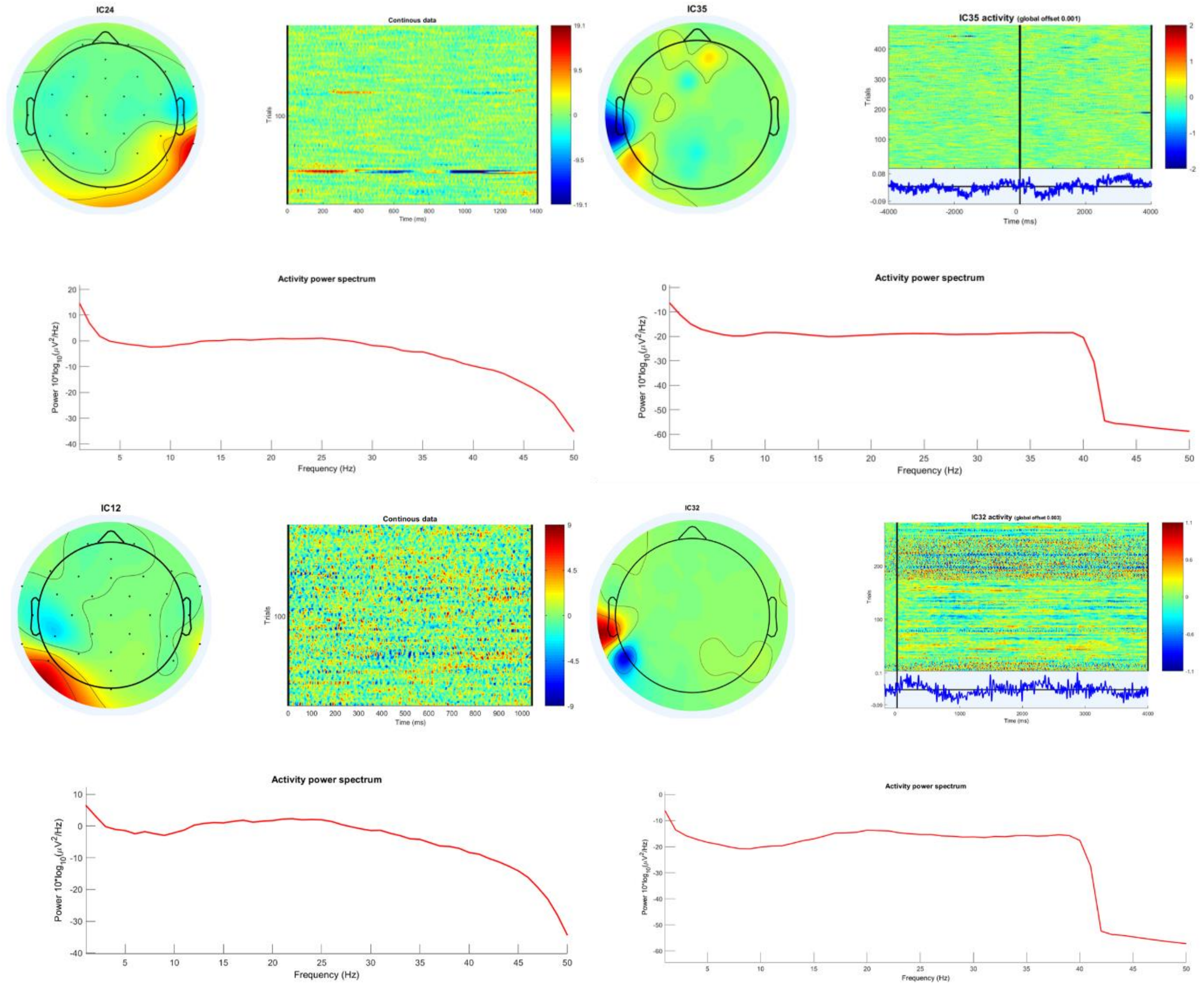

e. *(left column) Spectral Power plot of IC12 (bottom) and IC24 (top) in two different infants), representing extended long arm movements. (right column) Spectral power plot of an independent component representing two right-handed adult participants, each using a joystick in an unrelated experiment (author's own unpublished data).*

4 Supplementary Pilot Study 2 on Actively-Elicited Motion

This experiment aimed to replicate the EEG signal distortions described in the main manuscript using an active paradigm. A secondary goal was to generate a large number of repetitions of each motion type, and a long separate resting state recording, in a controlled environment where we purposefully aimed to elicit the desired motions by the infant, one at a time. Accordingly, one infant together with his mother, returned to the lab three times to perform a number of different tasks in order to elicit the selected motions from the first pilot, whilst EEG was recorded from both mother and infant. These motion-contaminated EEG recordings were then compared to their resting state EEG. In this experiment, we additionally asked the mother to generate the motions most frequently observed in the first behavioural pilot study. For the adult, we selected the following artifacts to be modelled: **Facial Movements** (**FMs**), comprising **Lip** and **Jaw** movements; **Limb Movements** (**LMs**), comprising **Hand** and **Arm** movements; and finally, **Postural Movements** (**PMs**), comprising **Up Neck** movements, **Nodding** (neck going down); **Leaning Forward** and **Leaning Back**. Although Lip, Neck Up and Nodding movements did not differ between the two play conditions observed in pilot study 1, they occurred over 10% of the time in the JP condition and therefore their effect on the adult's EEG signal was of interest. For the infant, **Facial Movements (FMs)** included **Talking/Babbling** and **Chewing** movements. **Limb Movements** (**LMs**) for the infant included **Hand**, **Arm**, **Foot** and **Leg**; and **Postural Movements** (**PMs**) for the infant were **Up Neck** movements and **Nodding**.

4.1 Methods

4.1.1 Participants

One male infant (18 months) and a female adult (his mother, aged 35 years) participated in the study. The adult was a native English speaker. She reported no neurological problems and normal hearing and vision for herself and for her infant.

4.1.2 Materials

The same objects and toys were used as in pilot study 1. The videos from first pilot were used as a reference to help the adult participant to imitate and reproduce the motions as accurately as possible.

4.1.3 Protocol

Modelling was performed by the adult and infant to generate multiple isolated and stereotypical exemplars of each motion.

The elicitation methods for Facial, Limb and Postural motions are summarised in Tables S6a and S6b for the infant and adult respectively, and a more detailed description is given below the tables. The infant's motions were elicited as he was interacting with objects or was watching a video. The infant's motions had a variable number of repetitions, ranging from 24 to 193 iterations (Table S6a), and varied more in length than the adults', lasting from just 0.25 seconds to tens of seconds (e.g. when the same motion, such as leg kicking, was continuously repeated). For motion artifact analysis, only periods with obvious head or body motions were selected. Insufficient Postural movements were elicited from the infant, and so no results are presented for this category of motion.

All motions were modelled by the adult participant with between 20 to 56 repetitions (Table S6b) and were recorded sequentially. Each adult motion lasted between 0.5 and 3-4 seconds.

Table S6a. Infant motion elicitation methods and number of repetitions

**Motion**	**Elicitation method**	**N reps**
**FACIAL MOTIONS (FMs)**		
Talking/Babbling	Soft toys and books were used to elicit talking	43
Chewing	Elicited using chewable toys or naturally occurring	45
**LIMB MOTIONS (LMs)**		
Hand	Using intricate toys to encourage small hand movements	116
Arm	Positioned toys so the infant reached out to retrieve them	193
Foot	Swept soft brushes and feathers over the infant's foot	24
Leg	Naturally occurring when infant kicked legs against table	99
**POSTURAL MOTIONS (PMs)**		
Leaning Forward	Slowly leaning forward in a chair	2
Leaning Back	Slowly leaning back from a leaning forward position	0

Table S6b. Adult motion elicitation methods and number of repetitions

Motion	Elicitation method	N reps
**FACIAL MOTIONS (FMs)**		
Lip Movement	Elicited through smiling and kissing actions	56
Jaw Movements	Elicited by silently mouthing through the vowels	22
**LIMB MOTIONS (LMs)**		
Hand	Using intricate toys to encourage small finger movements	35
Arm	Slow extensions of the arm forwards	38
**POSTURAL MOTIONS (PMs)**		
Neck Up	Slowly moving the head upwards, arching the neck up	27
Nodding	Slowly moving the head downwards, contracting the neck	20
Leaning Forward	Slowly leaning forward in a chair	23
Leaning Back	Slowly leaning back from a leaning forward position	22

Infant Artifact Modelling Descriptions

Talking

This was general infant communication towards the adult or an object. Given the typical age of the first pilot study's infants, noises considered talking comprised general babble. In order to replicate these noises, objects that the infant was known to expressly communicate with were used, including books and soft toys.

Chewing

This motion was predominantly observed when infants chewed his hand or toys during the sessions. For the purpose of this experiment, teething rings, and other 'chewable' toys were presented to the infant.

Whining

Whining was predominantly produced by the infant when he was fatigued or bored. This artifact was left until last to elicit, by which time the infant was starting to show signs of fatigue and frustration and produced whining noises.

Side-to-side movement

This movement, a relatively slow movement (not to be confused with a nod) was predominantly produced by the infants when looking around the room to observe objects or other adults. It was replicated by having two adults stand at some distance either side of the infant, holding a toy that

that produced a distracting noise. Each adult took a turn at distracting the infant, successfully creating a slow side-side movement.

Up-down movement

Similar to the aforementioned side-to-side, the up-down head movement motion was also relatively slow. It was noted, during both conditions in the first pilot study, that the infants often looked up towards the ceiling when bored and down towards the floor if a toy had been dropped. However, to elicit this motion an adult stood in front of the infant, and slowly raised and lowered their hand, containing a toy or object that sparked the infants interested to follow this motion.

Small hand

This artifact was most frequently produced, and often when the infant was interested in the texture of an object or toy, or an item that allowed them to weave their fingers in and out of. A Winkel toy (Figure S1) was observed as a toy seen to produce significant hand movements in pilot study 1 and was therefore chosen to elicit this artifact.

Small foot

This artifact was mostly observed when the infants flexed their feet. It primarily involved a rotating action from the ankle with a possible movement of toes. To elicit it we used a soft feather brush and gently swept it against the infant's toes.

Large arm

This artifact was primarily observed when an infant would extend their arm out to grab a toy, or to reach forward to the adult in the JP condition. In order to elicit this artifact, a toy that the infant had shown great interest in was put in front of the infant, just enough distance from him to create the same motion as observed in pilot study 1.

Large leg

This artifact was mostly observed by infants trying to stretch their legs or wriggle of the highchair, and most commonly produced during times of fatigue or frustration for the infant. This artifact was one of the final artifacts of the session to be modelled as fatigue and frustration would have likely set in by then, allowing for the elicitation of this artifact.

Adult Artifact Modelling Descriptions

Lip movement

This artifact predominantly was observed as a smiling or kissing action and was most frequently observed as a form of nonverbal communication from the adults to the infants. To elicit this artifact, the adult was requested to slowly produce separate smiling and kissing actions.

Up/down movements

This artifact was the same movement as outlined in the infant up-down movement. It was most commonly produced by the adults when trying to entertain the infant or attract their attention. To elicit this artifact, the adult was requested to slowly move their head up to the ceiling, then repeat the movement in a downwards action.

Jaw movements

This artifact involved a very animated jaw action, most commonly produced in order to attract the infant's attention or to entertain them. To replicate these, the adult was requested to silently mouth through the vowels. This produced jaw movements similar to those generated by the adult participants in first pilot.

Small hand

This artifact was the same movement as outlined in the infant's small hand movement description. The Winkel toy was used, which allowed the adult participant to replicate the same small hand movement as generated in the first pilot.

Large arm

This artifact was most commonly produced when the adult extended their arm to give the infant a toy, or to comfort them. This artifact was elicited by the adult participant slowly extending their arm forwards and backwards.

Leaning forward

This artifact was most frequently observed by the adult reaching forward to hold the infant's hand or to comfort them. To elicit this motion, the adult participant was requested to slowly lean forward in their chair towards an object.

Leaning back

This artifact was the opposing motion after the adult had leaned forward to comfort or interact with the infant. To produce this artifact, the adult participant was requested to slowly lean back in their chair from a leaning forward position. The adult actor was asked to repeat each single motion several times during a ten-minute period.

Resting State Description

The EEG during movement was compared with the EEG recording during a baseline (resting state (RS)) condition. The RS measurements were acquired from the adult in a separate continuous recording of 450 seconds during which the participant was instructed to relax with her eyes open and fixated on a cross at the centre of a laptop screen (Lenovo ThinkPad, 13", 1440 x 900 resolution, 15° angle), and to avoid any intentional movement. She was not instructed to avoid blinking or other reflex motions. Her feet, arms and shoulders were rested on cushions. For the infant, resting state measurements were obtained during periods of quiet relaxation, such as when sitting quietly in a high chair whilst watching a video. The total length of the concatenated separate resting state segments from the infant's recoding was 328 seconds.

4.1.4. Video and EEG acquisition

Video recordings. As in Studies 1 and 2, a Logitech High Definition Professional Web-camera was used to record the adult and infant (30 frames per second). Afterwards, each recording was manually coded to ascertain the exact start and end times of the modelled behaviours.

Video coding. The infant's and adult's motion timings, as well as the infant's resting state periods, were extracted from each video using an identical procedure as in the main experiment.

For the adult's resting state measurement, the video-coder confirmed that no overt motions were present at any time during the EEG recording.

Video-EEG synchronisation and EEG pre-processing. Both procedures were identical to the ones described in the main experiment.

4.1.5 Analysis of motion-related EEG artifacts

The infant's frequency bands were identical to the ones used in the descriptive statistics in the main experiment. The frequency bands for the adult data were Delta (1-3 Hz), Theta (3-7 Hz), Alpha (8-12 Hz), Low Beta (13-15 Hz), and High Beta (16-20 Hz).

In this analysis, we present the average power (amplitude squared) of the EEG signal in each frequency band, averaged over all epochs, for all electrodes.

4.2 Results

Examples of the raw EEG signal of artifacts produced by motion are provided in Figures S10 and S13 below.

4.2.1. Infant Raw EEG Examples

For the infant, some motion artifacts were not noticeably different to RS from inspection of the raw data. These include hand (Figure S10c) and arm (S10d) movements. Even for Talking/Babbling (S10e), the raw data is not obviously discernible from RS recording; this may be due to predominantly high-amplitude slow frequencies that are consistent with repetitive babbling jaw motions and dominating RS frequencies. Due to the similarity of these motion-contaminated recordings to RS, artefacts are much less likely to be excluded at pre-processing stage, either by eye, or by independent component analysis.

Figure S10. Infant raw EEG (filtered and average re-referenced) segment from continuous recording. All figures show 5 seconds of continuous recording.

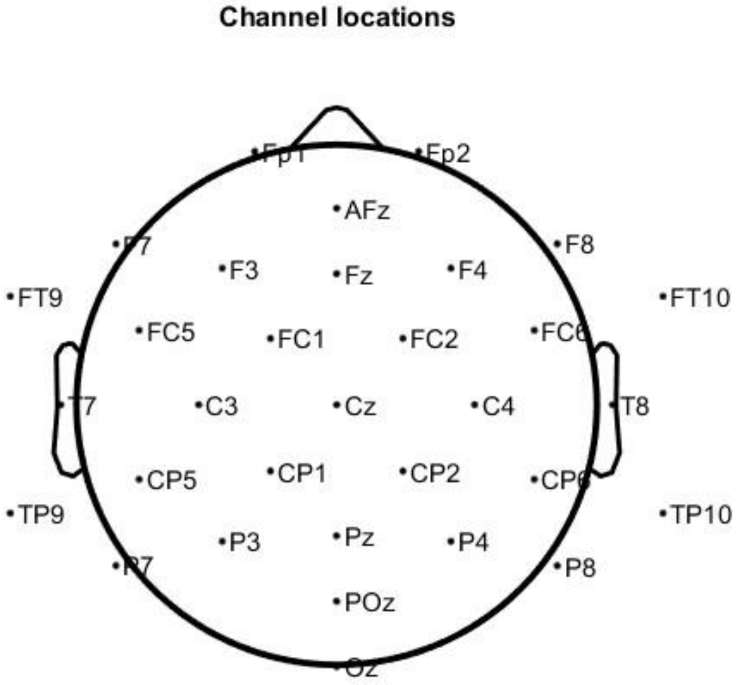

(a) Channel Locations

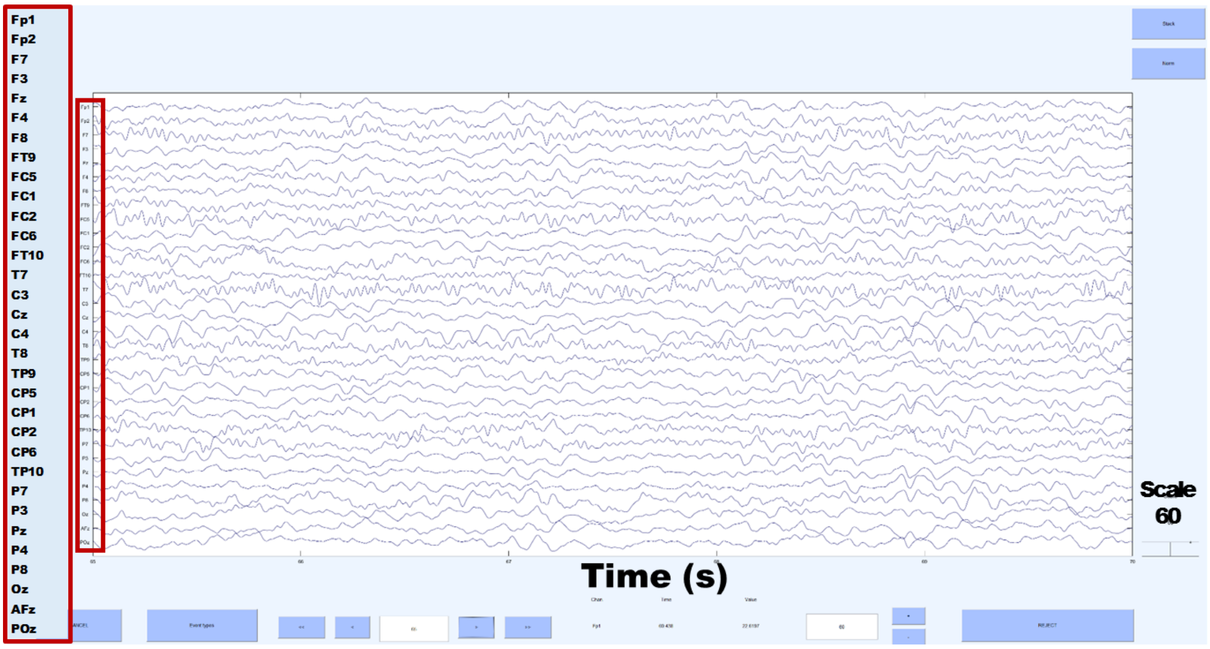

(b) Infant Resting State

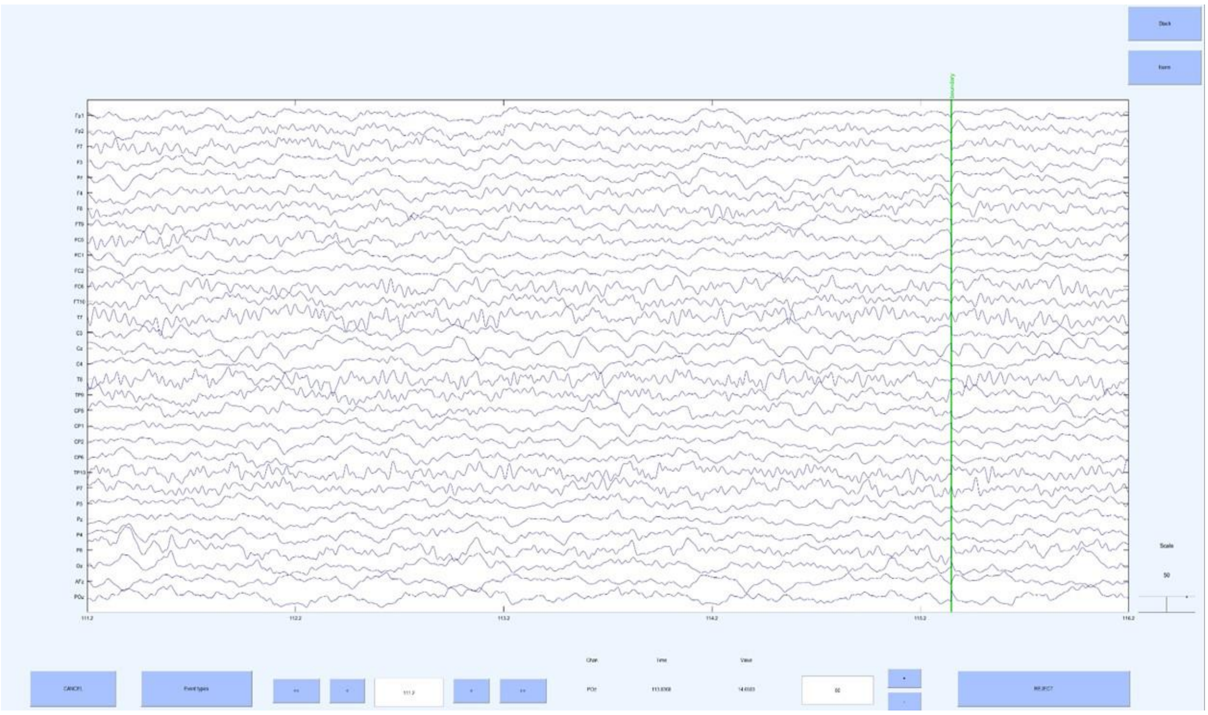

(c) Infant Limb Movements: Hand Movements

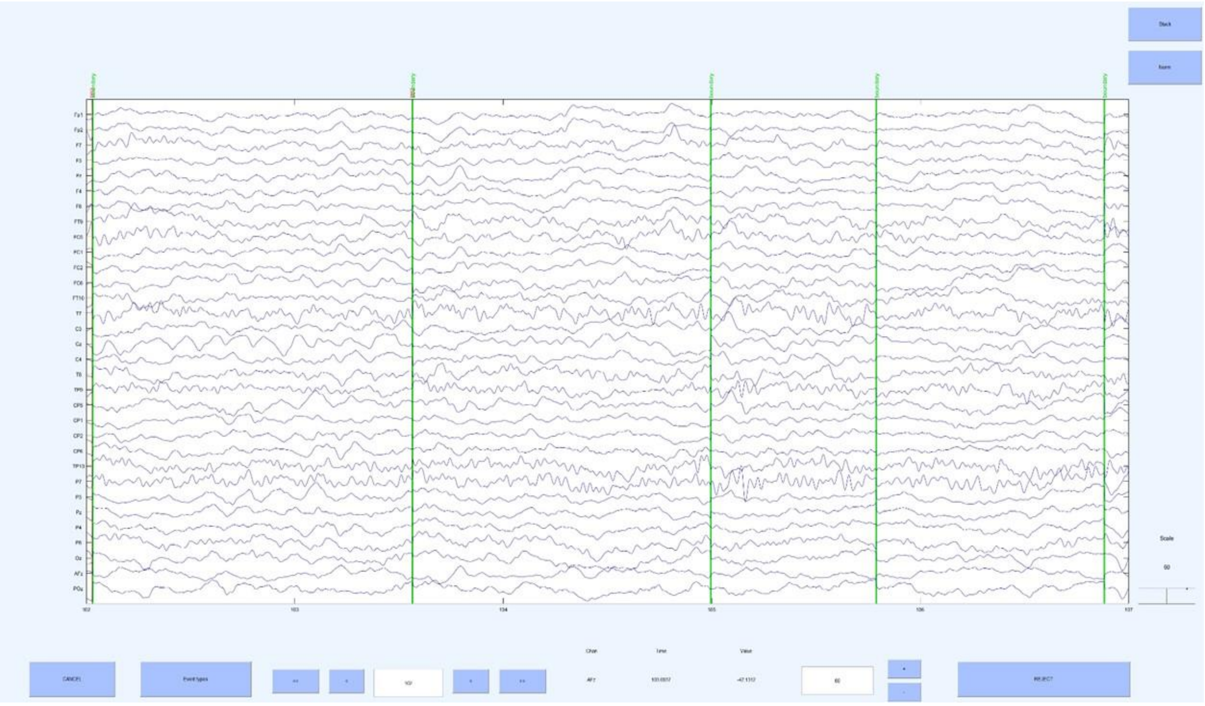

(d) Infant Limb Movements: Arm Movements

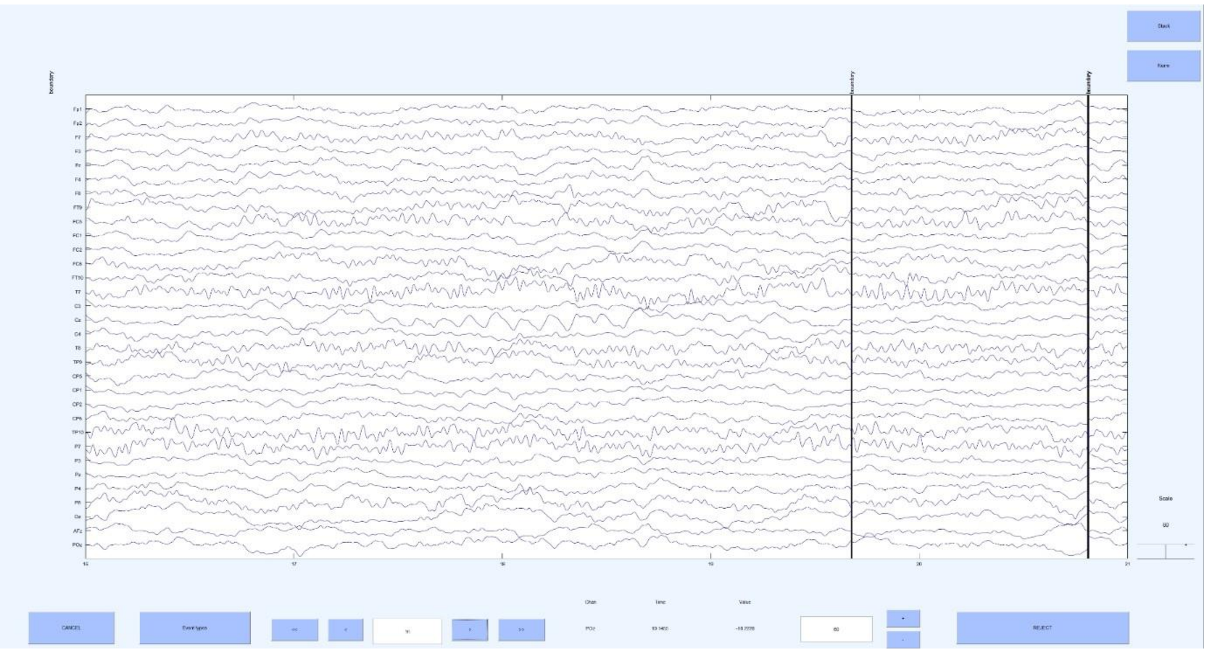

(a) Infant Head Movements: Talking/Babbling

4.2.2. Infant motion artifacts

4.2.2.1. Scalp topographies of infant resting state and motion artifacts

Resting state. The topology of infant resting state EEG was characterised by high alpha power across centro-parietal regions (Figure S11a), consistent with previous reports of the infant “mu rhythm” (Cuevas et al, 2014). We also observed strong power in the low and high beta bands over bilateral temporal regions, and in the delta band over posterior and midline electrodes. As these beta and delta activation patterns were also observed across all movement measurements (see Figure S11b, S11c), we inferred that these patterns reflected tonic (and not readily observable) muscular activity in our infant participant (i.e. jaw/neck tension).

Facial movements (talking/babbling, chewing). The two forms of facial movement produced highly similar scalp topographies (see Figure S11b).

Limb movements (hand, arm, foot, leg). Upper limb (hand and arm) movements produced larger decreases in central alpha power than lower limb (foot and leg) movements (see Figures S11c).

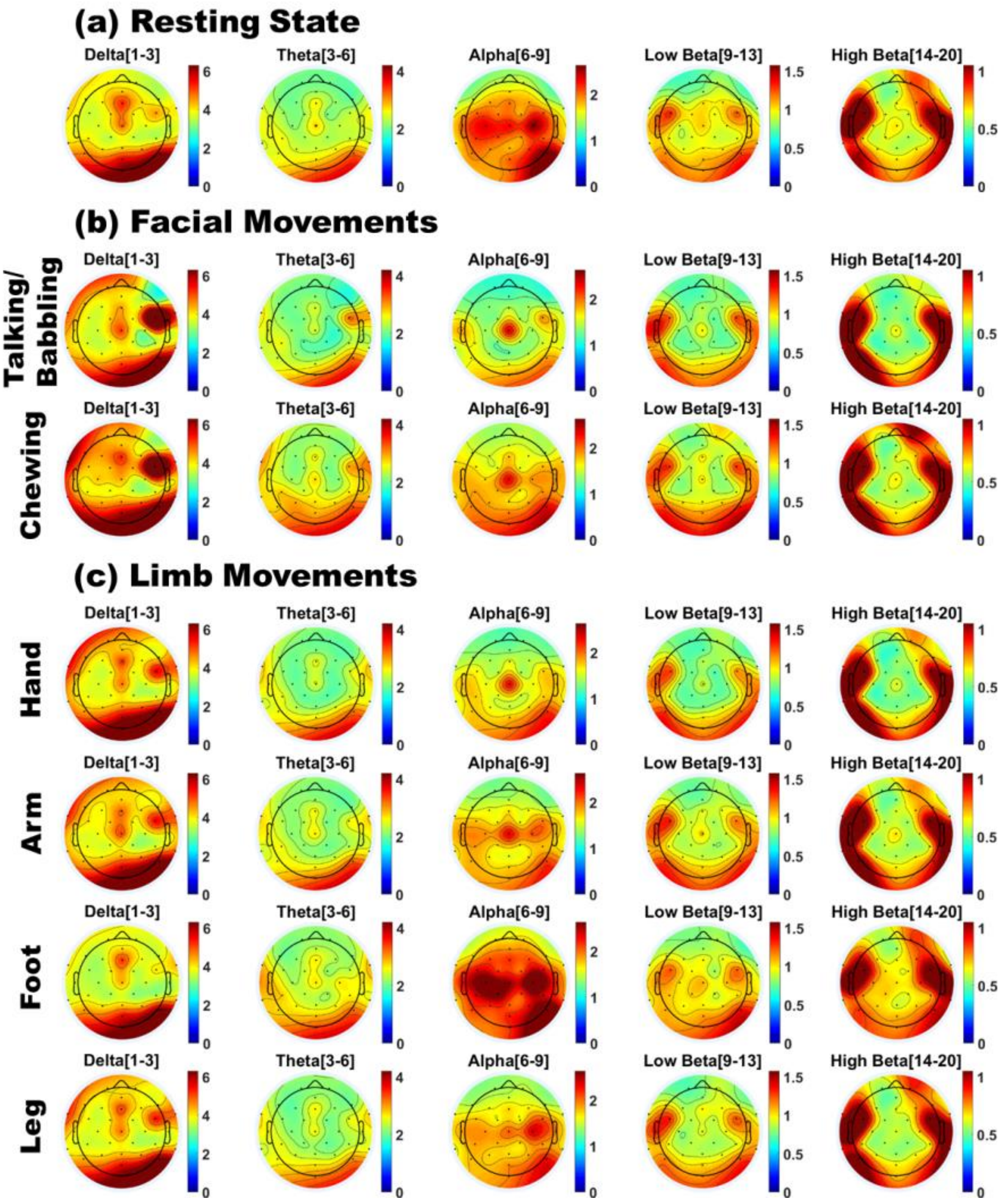

Figure S11. Scalp topographies of infant EEG power for (a) Resting state; (b) Facial movements; (c) Limb movements; Red indicates a region of high power, and blue indicates a region of low power.

4.2.4. Adult Raw EEG Examples

For the adult, the most notable distortions in the raw data were produced by Leaning Backwards and Forwards (*Figures S12 (b) and (c)*, respectively), possibly due to compromising the ground channel placed at the back of participant's neck. Raw amplitude during the presence of these artefacts was over 1000 uVs around the onset and the offset of the movement, which also led to high power in low frequencies in the subsequent FFT analysis. Other motions, such as Neck Up (*Figure S12d*), Nodding (*Figure S12e*), as well as Jaw related motions (*Figure S12f*), produced less prevalent, more short-lasting high-amplitude distortions peaking at 50-60 uVs that

might be detected in automatic or manual artifact rejection. However, for some motions, such as Hand Movements (*Figure S12h*) and Lip Movements (*Figure S12g*), artifacts were not noticeable at the raw data level even when time-locked to the onset of the movement and were much less likely to be excluded at the preprocessing stage.

Figure S12. Adult raw EEG (filtered and average re-referenced) segment from continuous recording. All figures show 5 seconds of continuous recording. Channels locations and order are identical to that for infant figures.

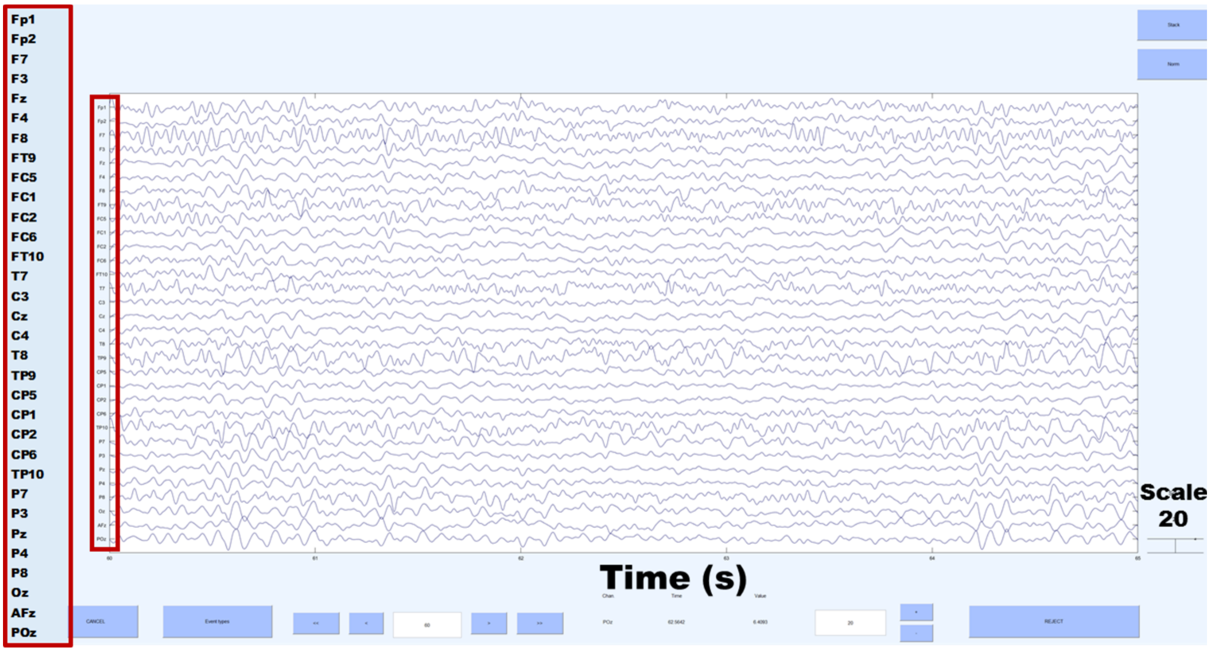

(a) Adult Resting State

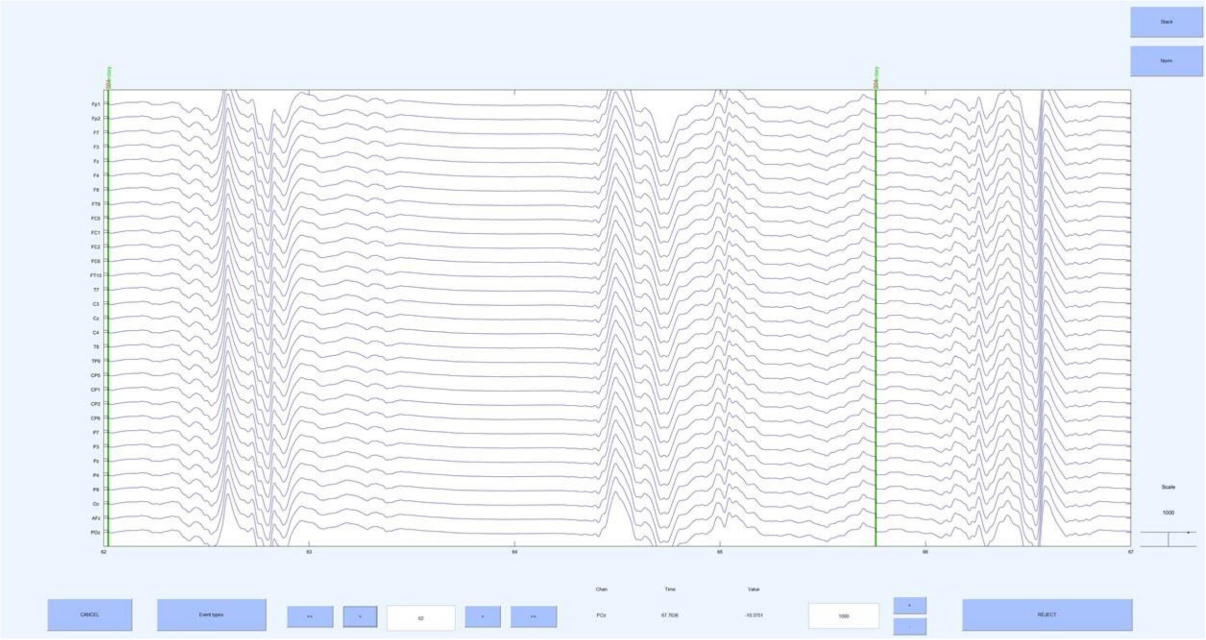

(b) Adult Postural Movements: Leaning Back

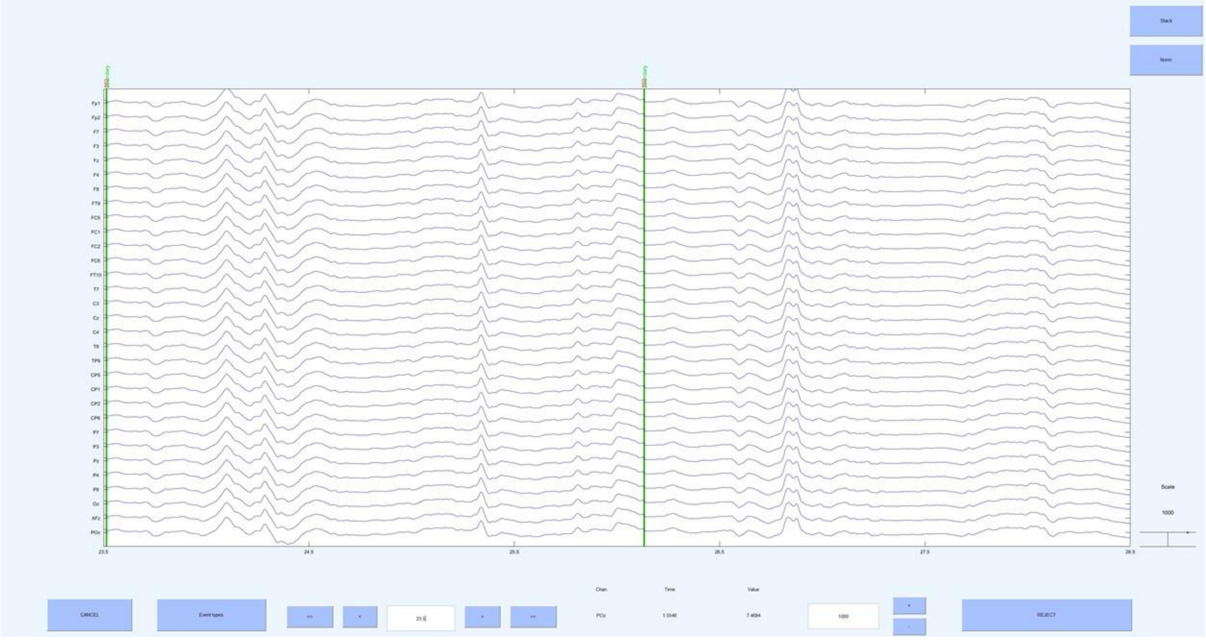

(c) Adult Postural Movements: Leaning Forward

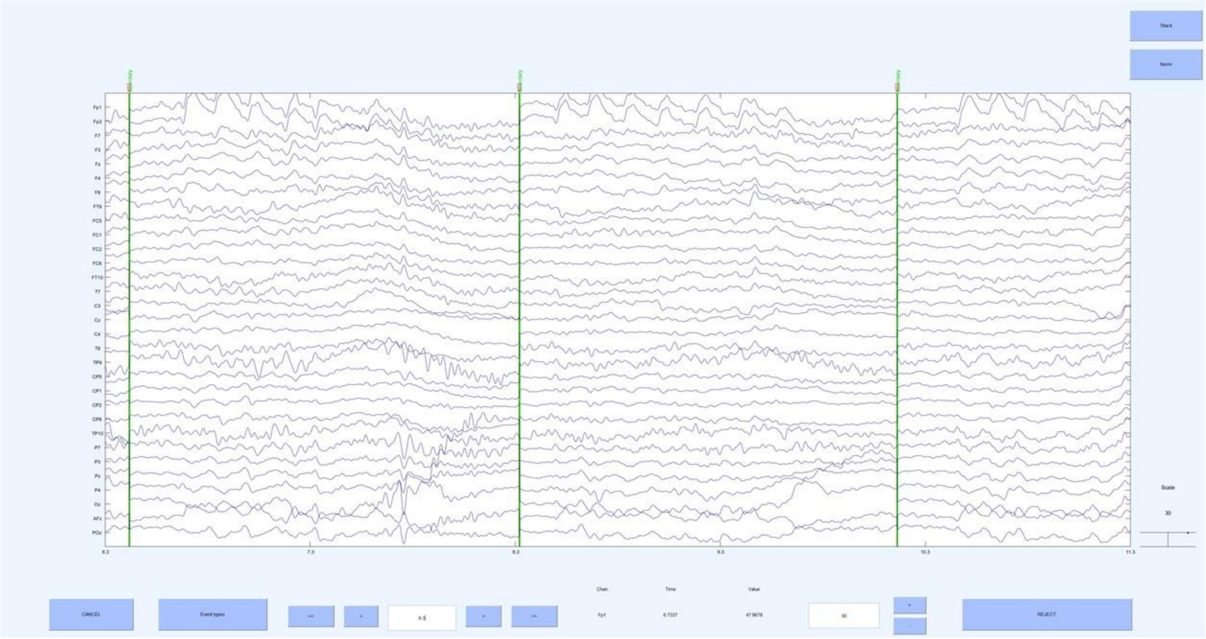

(d) Adult Postural Movements: Neck Up

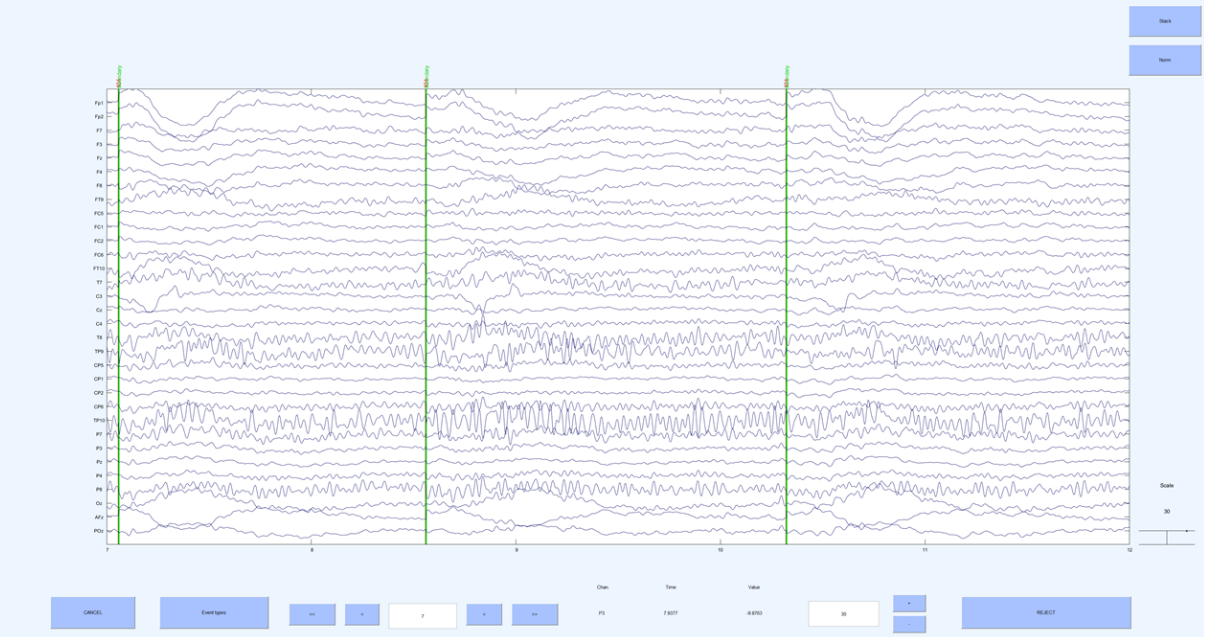

(e) Adult Postural Movements: Nodding

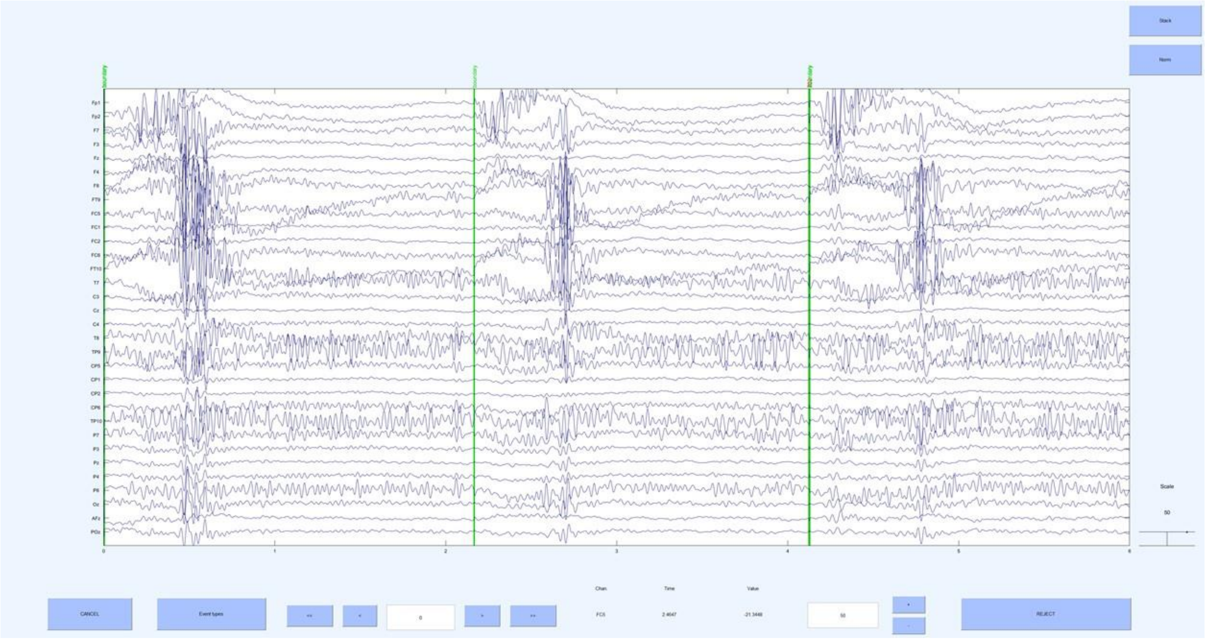

(f) Adult Facial Movements: Jaw Movements

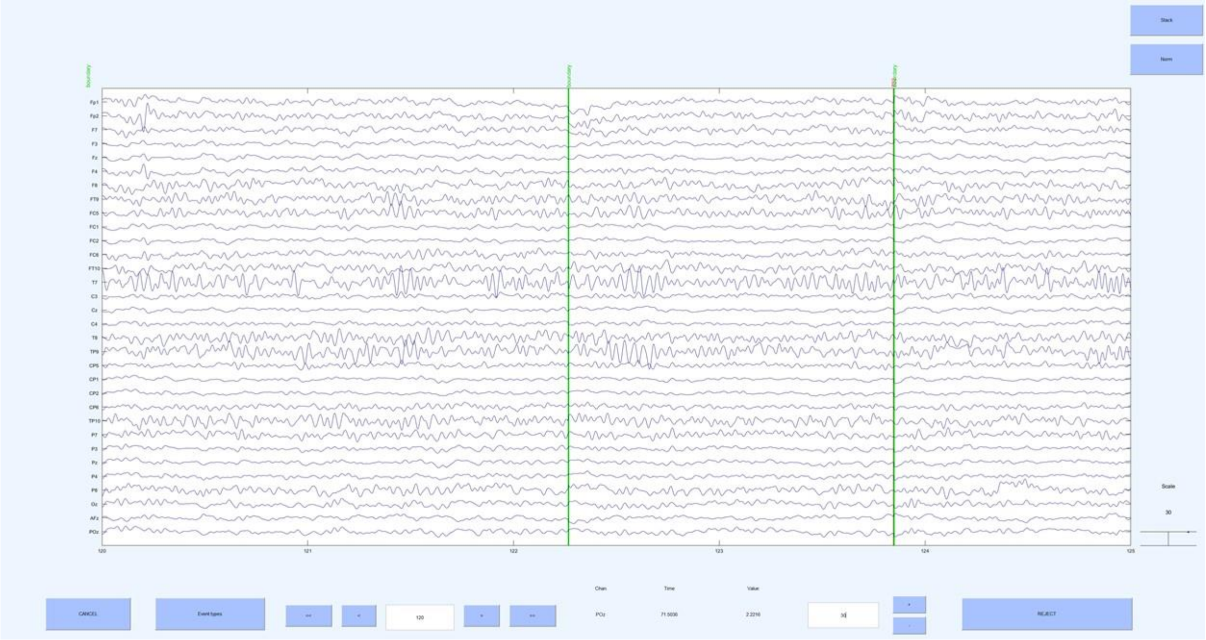

(g) Adult Facial Movements: Lip Movements

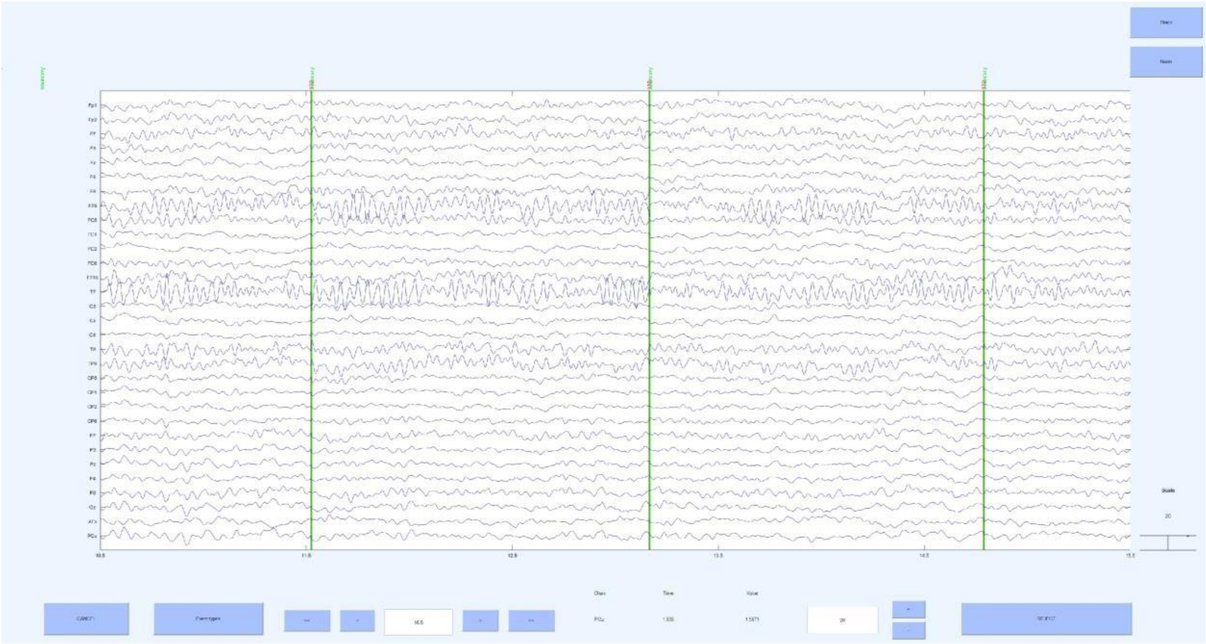

(h) Adult Limb Movements: Hand Movements

4.2.5. Adult motion artifacts

4.2.5.1. Scalp topographies of adult motion artifacts

Figure S13 shows the adult scalp EEG topographies for resting state power (Figure S13a) as well as for each motion artifact (S13b-d), in each frequency band. During resting state, we noted that the adult showed significant central midline theta neural activity, and also high power over frontal electrodes, particularly in the high beta band (15-20 Hz), which likely corresponded to oculomotor activity. This type of oculomotor activity can be removed from the data using the techniques (e.g. ICA) that we describe in the introduction.

Facial movements (lip, jaw). As shown in Figure S13b, both facial movements generated large increases in power over left and right temporal and parietal channels across all frequency bands, particularly in the delta and beta bands.

Limb movements (hand, arm). As shown in Figure S13c, limb movements produced relatively smaller power changes than facial movements.

Postural movements (neck up, nodding, leaning forward, leaning back). All postural movements produced large power increases across all frequency bands over frontal, occipital and parieto-occipital channels. Leaning Forwards and Leaning Back, in particular, produced very large power increases (over 50 uV^2/Hz across all bands), which arose because performing these motions involved synchronized displacement of the ground channel.

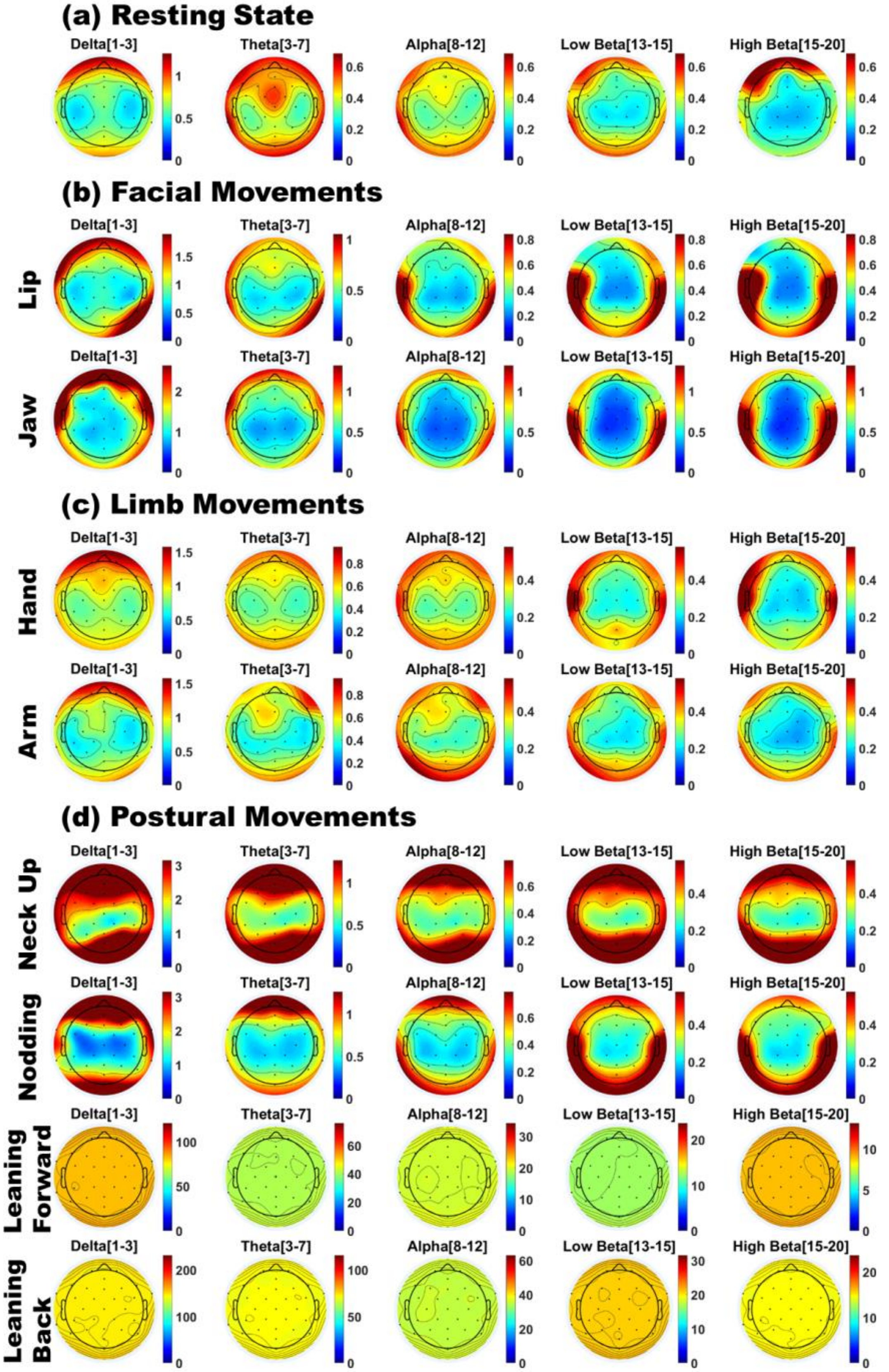

Figure S13. Scalp topographies of adult EEG power [uV^2/Hz] for (a) Resting state; (b) Facial movements; (c) Limb movements; and (d) Postural movements. Red indicates a region of high power, and blue indicates a region of low power.

BIBLIOGRAPHY

Rogers, J. C. & Davis, M. H. (2009) Categorical perception of speech without stimulus repetition in *Proceedings of the Interspeech meeting, Brighton, UK.*
